# Loss of adenomatous polyposis coli function renders intestinal epithelial cells resistant to the cytokine IL-22

**DOI:** 10.1371/journal.pbio.3000540

**Published:** 2019-11-26

**Authors:** Yu Chen, Maud Vandereyken, Ian P. Newton, Ignacio Moraga, Inke S. Näthke, Mahima Swamy

**Affiliations:** 1 Cell and Developmental Biology, School of Life Sciences, University of Dundee, Dundee, United Kingdom; 2 MRC Protein Phosphorylation and Ubiquitylation Unit (PPU), School of Life Sciences, University of Dundee, Dundee, United Kingdom; 3 Cell Signalling and Immunology, School of Life Sciences, University of Dundee, Dundee, United Kingdom; B.C. Cancer Agency, CANADA

## Abstract

Interleukin-22 (IL-22) is a critical immune defence cytokine that maintains intestinal homeostasis and promotes wound healing and tissue regeneration, which can support the growth of colorectal tumours. Mutations in the adenomatous polyposis coli gene (*Apc*) are a major driver of familial colorectal cancers (CRCs). How IL-22 contributes to APC-mediated tumorigenesis is poorly understood. To investigate IL-22 signalling in wild-type (WT) and APC-mutant cells, we performed RNA sequencing (RNAseq) of IL-22–treated murine small intestinal epithelial organoids. In WT epithelia, antimicrobial defence and cellular stress response pathways were most strongly induced by IL-22. Surprisingly, although IL-22 activates signal transducer and activator of transcription 3 (STAT3) in APC-mutant cells, STAT3 target genes were not induced. Our analyses revealed that *Apc*^*Min/Min*^ cells are resistant to IL-22 due to reduced expression of the IL-22 receptor, and increased expression of inhibitors of STAT3, particularly histone deacetylases (HDACs). We further show that IL-22 increases DNA damage and genomic instability, which can accelerate cellular transition from heterozygosity (*Apc*^*Min/+*^) to homozygosity *(Apc*^*Min/Min*^) to drive tumour formation. Our data reveal an unexpected role for IL-22 in promoting early tumorigenesis while excluding a function for IL-22 in transformed epithelial cells.

## Abstract 2

The adenomatous polyposis coli (APC) gene is mutated in 85% of colorectal cancers. This study shows that when APC is mutated in murine intestinal epithelial cells, they no longer respond to IL-22, a cytokine that is considered important for colorectal cancer progression; this has implications for IL-22 as a therapeutic target for cancer treatment.

## Introduction

Chronic inflammation is positively associated with intestinal tumorigenesis [1,2]. Recent data have particularly implicated the cytokine Interleukin-22 (IL-22) in colorectal cancers (CRCs). Consistently, IL-22 producing cells are enriched in human CRC and associated with its development [3–5]. IL-22 is produced by type 3 innate lymphoid cells (ILCs), CD4^+^ and CD8^+^ T cells, and γδ T cells during intestinal inflammation [4,6–9]. IL-22 binds to the IL-22 receptor, which is composed of two heterodimeric subunits, the interleukin 22 receptor subunit alpha 1 (IL-22RA1) and the interleukin 10 receptor 2 (IL-10R2) [10,11]. In the intestine, IL-22 receptor expression is restricted to non-haematopoietic cells, particularly intestinal epithelial cells [12]. IL-22 activates the signal transducer and activator of transcription 3 (STAT3) pathway and regulates intestinal epithelial functions, including tissue repair, mucus secretion, and antimicrobial activity [7,12–14]. Increasing evidence indicates that IL-22 not only facilitates tissue protection but also has prosurvival and proliferative effects that can promote CRC [8,15–18].

Inactivating mutations in the tumour suppressor adenomatous polyposis coli gene (*Apc*) cause intestinal tumorigenesis and are a major cause of hereditary CRC [19], a condition known as familial adenomatous polyposis (FAP). In FAP patients, adenomatous polyps are present throughout the colon and develop into cancer if left untreated. In addition to FAP, inactivating mutations in the *Apc* gene are present in more than 80% of nonhereditary CRCs [20]. APC is best known as a negative regulator of Wnt signalling, contributing to regulation of cell proliferation and differentiation [21,22]. The *Apc*^*Min/+*^ (multiple intestinal neoplasia [Min]) mice mimic FAP intestinal tumorigenesis and carry a truncated, non-functional version of the *Apc* gene on one allele. Spontaneous loss of heterozygosity (LOH) in intestinal epithelial cells leads to loss of the wild-type (WT) *Apc* allele (*Apc*^*Min/Min*^ genotype). The resulting increased Wnt signalling and other epithelial changes together lead to adenoma (polyp) formation in the intestine. In this and other *Apc*-deficient mouse models, inflammatory cytokines are established augmenting factors for intestinal cancer development [23,24]. Consistent with the idea that IL-22 might also play a role in this process, *Il22*^−/−^*Apc*^*Min/+*^ mice develop fewer and smaller tumours than *Il22*^*+/+*^*Apc*^*Min/+*^mice [16]. However, it is not clear how IL-22 impacts on APC-mutant cells to increase tumorigenesis.

The aim of our study was to understand how IL-22 contributes to intestinal tumorigenesis in *Apc*^*Min/+*^ mice and determine whether loss of APC function affects the cellular response to IL-22. To this end, we performed RNA sequencing (RNAseq) on intestinal organoids from WT and *Apc*^*Min/+*^ mice composed of *Apc*^*Min/Min*^ cells to measure intestinal epithelial responses to IL-22 stimulation. Organoids are primary intestinal epithelial cell cultures that grow in 3 dimensions [25]. Importantly, organoids contain only intestinal epithelial cells–including stem cells, Paneth cells, enteroendocrine cells, goblet cells, and enterocytes but no immune or stromal cells, allowing us to identify the direct effects of IL-22 on small intestinal epithelia. We found that, surprisingly, APC-deficient cells were resistant to stimulation by IL-22. This was due to both reduced expression of the IL-22 receptor and high expression of negative regulatory histone deacetylases (HDACs). Conversely, *Apc*^*Min/+*^ heterozygous cells responded to IL-22 similarly to WT cells by up-regulating cellular stress responses involved in the production of reactive nitrogen species. This stress response correlated with enhanced DNA damage, which in turn correlated with the accelerated transformation of *Apc*^*Min/+*^ cells to *Apc*^*Min/Min*^ genotype in the presence of IL-22, which ultimately leads to development of adenomas.

## Materials and methods

### Ethics statement

All animal studies and breeding were approved by the University of Dundee ethical review committee and were performed under a UK Home Office project license (project licenses PPL60/4172, 70/8813, and PD4D8EFEF) in accordance with the Animal Scientific Procedures Act (1986). All study plans were approved by the Named Veterinary Surgeon and Compliance Officer of the Biological Resource Facility, University of Dundee.

### Mice and treatment

Healthy C57BL/6J WT male or female adult mice between 60 and 120 days old bred in-house were used for preparing WT organoids. For *Apc*^*Min/Min*^ organoids or *Apc*^*Min/+*^ organoids, *Apc*^*Min/+*^ adult (male or female) mice aged approximately 90 days or 60 days, respectively, were used. Mice were maintained in a standard barrier facility on a 12-hour light/dark cycle at 21°C, 55%–65% relative humidity. Mice tested negative for all pathogens on the current FELASA list, except for *Helicobacter* spp. Mice were maintained in standard individually ventilated cages with Eco-Pure Chips 6 and were fed an autoclaved R&M3 diet (Special Diet Services, Essex, UK) and autoclaved water ad libitum, and cages were changed at least every 2 weeks.

For short-term IL-22 treatment, WT or *Apc*^*Min/+*^ mice aged 90 days were injected intraperitoneally with 1 μg IL-22, and PBS-injected mice were used as a nonstimulated control. Mice were sampled 1–24 hours after treatment. For longer-term treatments, WT or *Apc*^*Min/+*^ mice aged 28–35 days were injected intraperitoneally with 1 μg of IL-22 or PBS or were left untreated, twice every week for 4 weeks. One week after the last injection, intestines were harvested, and polyp load was assessed.

### Intestinal organoid culture and treatment

Small intestines of C57BL/6J mice were longitudinally opened and washed with PBS. Villi were removed by scraping with a glass coverslip. Following further PBS washes, small intestinal tissue was incubated in 3 mM EDTA in PBS at 4°C for 20 minutes. After being shaken for 1 minute, the tissue was removed, and the crypts were collected by centrifugation at 100*g* for 3 minutes at 4°C. Crypts were washed with PBS and incubated with TrypLE Express (Thermo Fisher Scientific, Waltham, MA) at 37°C for 5 minutes. Cells were suspended in Advanced DMEM/F12 (ADF) medium (Thermo Fisher Scientific) and were passed through a 40 μm cell strainer (Greiner, Kremsmunster, Austria). After centrifugation at 2,000 rpm for 3 minutes, cells were resuspended in Matrigel (Corning, NY) and were plated onto 24-well plates. For WT or *Apc*^*Min/+*^ organoid culture, cells were incubated in a 37°C 5% CO_2_ incubator for 15 minutes and were then supplemented with crypt medium (ADF medium supplemented with HEPES [Thermo Fisher Scientific], GlutaMax-1 [Thermo Fisher Scientific], N-acetylcysteine [10 mM, Sigma, St. Louis, MO], N2 [Thermo Fisher Scientific], B27 [Thermo Fisher Scientific]; 100 U/mL penicillin/100 μg/mL streptomycin [Thermo Fisher Scientific]) that was further supplemented with EGF (50 ng/mL, Thermo Fisher Scientific), Noggin (100 ng/ml, Peprotech, London, UK), R-Spondin–conditioned medium, valproic acid (VPA; 1 μM, Sigma), and ROCK Inhibitor Y27632 (Sigma). R-Spondin–conditioned medium [26] was produced from stably transfected L-cells (gift from Owen Sansom). After 48-hour incubation, medium was replaced by crypt medium supplemented with EGF, Noggin, and R-Spondin. For *Apc*^*Min/Min*^ organoid culture, crypt medium containing EGF, Noggin, and R-Spondin was used for the first 48 hours. *Apc*^*Min/Min*^ organoids were then cultured in crypt medium alone for a week before the medium was replaced with crypt medium supplied with EGF, Noggin, and R-Spondin. Medium was replaced every 2 days. Organoids were passaged approximately every 4 to 6 days. Organoids were used for experiments at day 3 after passaging. For IL-22 stimulation experiment, organoids were treated with IL-22 (Peprotech, London, UK). Concentration and duration are described in the figure legends. For HDAC inhibition, organoids were incubated with sodium butyrate (NaBu) (1 mM; Sigma, St. Louis, MO), VPA (1 μM, Sigma, St. Louis, MO), or Trichostatin A (TSA; 50 nM, Sigma) for 16 hours before stimulation with IL-22 (10 ng/mL) along with media containing the corresponding HDAC inhibitor for 3 hours.

### Immunohistochemistry

For immunohistochemistry (IHC), small intestine was removed and fixed in 4% PFA at 4°C overnight. After processing and paraffin embedding, specimens were cut into 5 μm sections and placed onto SuperFrost Plus Adhesion Slides (Thermo Fisher Scientific, Waltham, MA) and dried at 60°C overnight. Slides were deparaffined with Histo-Clear (National Diagnostics) and dehydrated through graded ethanol solutions. For antigen retrieval, EDTA buffer (1 mM EDTA, 0.05% Tween 20 [pH 8]) was used for phosphorylated STAT3 (pSTAT3) and β-catenin costaining, and sodium citrate buffer (10 mM sodium citrate, 0.05% Tween 20 [pH 6.0]) for RegIIIγ and β-catenin costaining. Slides were rinsed in PBS and incubated with blocking buffer 1 (PBS, 2% BSA) for 15 minutes, followed by incubation with blocking buffer 2 (PBS, 1% BSA, 0.3% Triton) for 15 minutes. Slides were covered with primary antibodies diluted in blocking buffer 2 for 3 hours. Primary antibodies used included anti-β-catenin (1:200, BD, 610154), pSTAT3 (Tyrosine 705) (1:200, Cell Signaling, Danvers, MA, 9145), anti-RegIIIγ (1:200; gift from Lora Hooper), anti-HDAC1 (1:400, Cell Signaling, Danvers, MA, 34589), and anti-HDAC2 (1:1000, Cell Signaling, Danvers, MA, 5113). After TBST (TBS, 0.1% Tween-20) washes, slides were incubated with Alexa Fluor conjugated secondary antibody (1:500, Thermo Fisher Scientific, Waltham, MA) and DAPI (1 μg/ml, Thermo Fisher Scientific, Waltham, MA) for 30 minutes. After TBST washes, the cover slides were mounted on glass slides with Prolong Gold Antifade media (Thermo Fisher Scientific). Images were analysed in Imaris software (Bitplane, Zurich, Switzerland) or Fiji software [27].

### Immunofluorescence staining of intestinal organoids

For imaging and immunofluorescence, organoids were grown in Matrigel on 8-well μ-slides (Ibidi, Grafelfing, Germany). Organoids were fixed in prewarmed (37°C) 4% paraformaldehyde (pH 7.4; Sigma, St. Louis, MO) for 20 minutes at 37°C. Organoids were permeabilized by using permeabilization buffer (PBS, 1% Triton X-100) for 1 hour, followed by blocking step with blocking buffer (PBS, 1% BSA, 3% normal goat serum, 0.2% Triton X-100) for 1 hour at room temperature. Organoids were then incubated with primary antibody diluted in working buffer (PBS, 0.1% BSA, 0.3% normal goat serum, 0.2% Triton X-100) overnight at room temperature. Primary antibodies used were as follows: anti-Phospho-Stat3 (Tyrosine 705) (1:100, Cell Signaling, Danvers, MA, 9131), anti-Phospho-Histone H2A.X (Serine139) (also known as γH2AX), 1:200, Cell Signaling, 9718), and anti-IL22RA1 (1:200, Thermo Fisher Scientific, 496514). After washes with working buffer, organoids were incubated with Alexa Fluor conjugated secondary antibody (1:500, Thermo Fisher Scientific) and Hoechst 33342 (5 μg/mL; Thermo Fisher Scientific) overnight at room temperature. Organoids were then washed with working buffer and were mounted with ProLong Gold antifade mountant (Thermo Fisher Scientific). Immunofluorescence was visualized under Zeiss 710 confocal microscope. Images were analysed by using Imaris software (Bitplane, Zurich, Switzerland).

### Western blotting

Organoids were washed with ice-cold PBS, and cell pellets were lysed in lysis buffer (40 mM Tris-HCl [pH 7.5], 120 mM NaCl, 0.27M sucrose, 1 mM EDTA [pH 8], 1 mM EGTA, 10 mM β-glycerophosphate, 5 mM sodium pyrophosphate, 1% Triton X-100) containing protein and phosphatase inhibitors. Proteins were separated on NuPAGE 4%–12% Bis-Tris Protein Gels (Thermo Fisher Scientific) and were transferred to Protran nitrocellulose membrane (0.2 μm‎, GE Healthcare, Chicago, IL). Immunoblotting was performed with primary antibodies diluted in 5% BSA/TBS-Triton overnight at 4°C. Primary antibodies used were as follows: anti-Phospho-Stat3 (Tyrosine 705) (1:2000, Cell Signaling, Waltham, MA, 9131), anti-Stat3 (1:2000, Cell Signaling, 9139), anti-Phospho-Stat3 (Serine 727) (1:1000, Cell Signaling, 9134), anti-Phospho-Stat1 (Tyrosine 701) (1:1000, Cell Signaling, 9167), anti-Stat1 (1:2000, BD, Franklin Lakes, NJ, 610185), anti-TBP (1:2000, Proteintech, Chicago, IL, 66166-1-lg), anti-HDAC1 (1:1000, 06–720, Millipore, Burlington, MA), anti-HDAC2 (1:1000, Cell Signaling, 5113), anti-inducible nitric oxide synthase (iNOS) (1:200, Abcam, Cambridge, UK, ab15323), and anti-phospho H2AX (Serine 139) (γH2AX, 1:500, Biolegend, San Diego, CA, 613401). Blots were incubated with IRDye800/700-conjugated secondary antibodies (Rockland, Pottstown, PA, 1:5000) for 1 hour at room temperature. Proteins were detected with LiCor Odyssey Imager (Licor Biosciences, Lincoln, NE. Western blots were analysed in Image Studio Lite 5.2 software (Licor Biosciences).

### Quantitative real-time reverse transcription polymerase chain reaction

Total RNA was isolated from organoids by using NucleoSpin RNA Isolation Kit (Macherey-Nagel, Duren, Germany). cDNA was prepared from total RNA by using qScript cDNA Synthesis Kit (QuantaBio, Beverly, MA). Real-time polymerase chain reaction (PCR) was performed by using PerfeCTa SYBR green FastMix for iQ (QuantaBio) in a quantitative PCR (qPCR) machine (CFX-Connect Real-Time System, Bio-Rad, Hercules, CA). Primers (Eurofins MWG, Ebersberg, Germany) used are shown in S1 Table.

### RNAseq

WT or *Apc*^*Min/Min*^ organoids were stimulated with IL-22 (2 ng/mL) for 3 hours. Total RNA was isolated from organoids by using NucleoSpin RNA isolation Kit (Macherey-Nagel). RNAseq was performed by the Finnish Microarray and Sequencing Centre and analysed by the Bioinformatics Unit, Turku Centre for Biotechnology, Finland. In brief, RNA was prepared for the sequencing using Illumina TruSeq Stranded mRNA sample Preparation Kit. mRNA was sequenced with the HiSeq 2500 instrument using single-end sequencing chemistry and 50 bp red length. STAR version 2.5.2b was used to align the reads against the reference genome. The “Subreads” package (version 1.5.1) was then used to associate reads with known genes and to count the number of reads associated with each gene. The read counts were normalised using the TMM normalisation method of the edgeR R/Bioconductor package. The TMM normalised counts are represented as Reads Per Kilobase of exon per Million reads mapped (RPKM) values.

For group comparison, statistical testing between paired samples in the groups was performed in R package Limma (Bioconductor). For functional analysis, topGO and GOstats packages in R/Bioconductor were used for the enrichment analysis of differentially expressed filtered gene lists. Further analyses were performed using the DAVID Functional Annotation tools (https://david.ncifcrf.gov). Heatmaps were prepared using Morpheus software (https://software.broadinstitute.org/morpheus) and the Viridis colour palette. Fully annotated data from each comparison are available in S2–S5 Tables. The complete raw and normalized data are available in the NCBI Gene Expression Omnibus database (https://www.ncbi.nlm.nih.gov/geo; accession number GSE139332).

### Flow cytometry

To prepare single cell suspensions, organoids were dissociated by incubation with TrypLE Express at 37°C for 5 minutes. Cells were collected by centrifugation at 2,000 rpm for 2 minutes. For IL-22RA1 staining, cells were stained with IL-22RA1 antibody (1:100, Proteintech, Chicago, IL, 13462-1-AP) for 30 minutes on ice. After being washed with staining buffer (PBS, 1% FBS, 2 mM EDTA), cells were incubated with Donkey Anti-Rabbit Alexa Fluor 647 antibody (1:500, ThermoFisher Scientific) for 30 minutes on ice. After being washed with staining buffer, cells were resuspended in staining buffer containing DAPI (2.5 μg/mL, Thermo Fisher Scientific).

For STAT3 staining, after incubation with TrypLE Express, single cells were incubated in 0.5 mL PBS containing LIVE/DEAD Fixable Blue Dead Cell Stain kit (1:1000, Thermo Fisher Scientific) on ice for 10 minutes. Cell were washed with staining buffer and then collected by centrifugation at 2,000 rpm for 2 minutes. Cells were fixed with 3 mL prewarmed 0.5% PFA/PBS at 37°C for 15 minutes and then washed with staining buffer. Permeabilization was performed by incubation with ice-cold 70% ethanol on ice for 10 minutes. After being washed with staining buffer, cells were stained with STAT3 antibody (1: 200, Cell Signaling, 9139) for 1 hour. After being washed with staining buffer, cells were stained with goat anti-mouse Alexa 568 antibody (Thermo Fisher Scientific) for 1 hour. After being washed with staining buffer, cells were resuspended in 500 μl staining buffer and analysed by LSRFortessa Cell Analyzer (BD, Franklin Lakes, NJ). Flow cytometry data were analysed by using FlowJo software version 9 (FlowJo, LLC, Ashland, OR).

### Statistical analysis

All statistical analyses were performed using Prism 7 (GraphPad, San Diego, CA). Methods of comparison are stated in figure legends, and normal distribution was assumed for all reverse transcription (RT)-qPCR data. For tumour numbers, normality and equal standard deviation were checked using the Shapiro-Wilks test and the Welch’s test, respectively. At least 3 independent experiments were performed for each data set. Mean ± standard deviation is shown in every figure.

## Results

### The IL-22 regulated transcriptome in small intestinal epithelial cells

**Fig 1:**
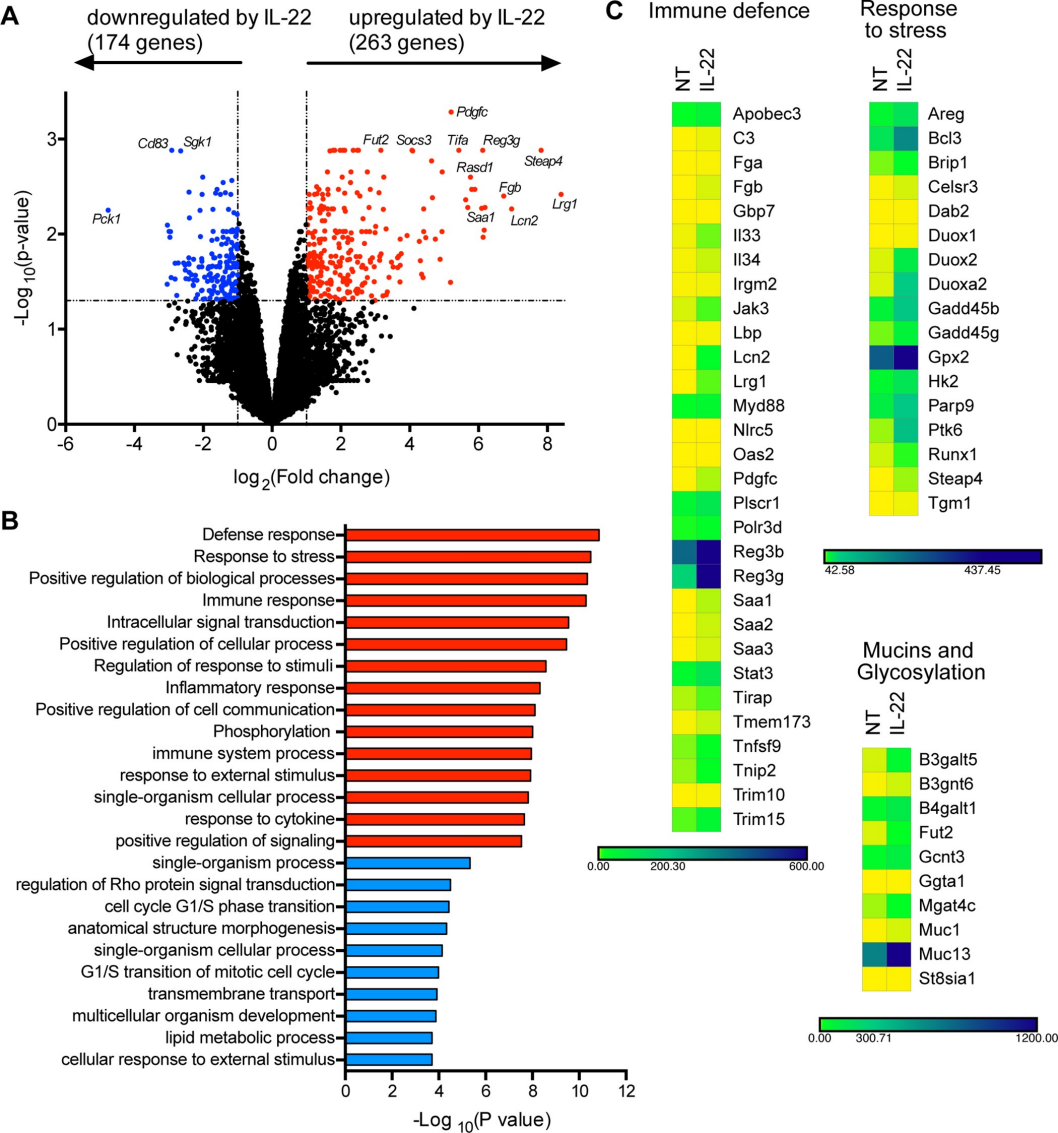
IL-22 regulates genes involved in intestinal immune defence responses. WT organoids were treated with IL-22 (2 ng/ml) for 3 hours. RNA was isolated for RNAseq analysis. (A) Volcano plot showing fold change (log_2_) in gene expression of WT organoids treated with IL-22 (2 ng/ml) for 3 hours compared to untreated WT organoids, plotted against significance (−log_10_[*p*-value]). Red = significantly up-regulated gene (fold change > 2, *p* < 0.05), blue = significantly down-regulated genes (fold change < 0.5, *p* < 0.05). (B) Functional enrichment analysis (GO term: “Biological processes”) for genes up- or down-regulated in WT organoids treated with IL-22, compared to untreated WT organoids. Shown are the top 15 up-regulated (red) and 10 down-regulated pathways (blue). (C) Heatmaps displaying mRNA expression level of significantly regulated genes in WT organoids either not treated or treated with IL-22. Genes of interest were manually grouped by their main biological function. Numerical values for panel B are available in S1 Data and for panels A and C in S2 Table. GO, gene ontology; IL-22, interleukin-22; NT, not treated; RNAseq, RNA sequencing; WT, wild-type.

IL-22 regulates multiple genes involved in antimicrobial defence, proliferation, and immune signalling. However, this varies according to cell type [14,28,29], and the effects of IL-22 on primary small intestinal epithelial cells have not yet been globally evaluated. To identify the responses of small intestinal epithelial cells to IL-22, we derived primary organoid cultures from WT murine small intestines and treated them with IL-22. We performed deep RNAseq on WT small intestinal organoids 3 hours after treatment with IL-22 to measure the global IL-22–regulated transcriptome. We chose this time point because we were interested in the immediate early effects of IL-22. The results showed that IL-22 significantly affected expression of 437 genes in WT organoids (Fig 1A and S1A Fig). Overwhelmingly, most of these were involved in immune response and defence against infection and stress (Fig 1B and 1C). These included genes with direct antimicrobial activity (e.g., *Reg3g*, *Lcn2*), genes involved in pathogen recognition and immune signalling (e.g., *Tmem173* [STING1], *Myd88*), genes involved in mucus production and mucin glycosylation (e.g., *Fut2*, *B4galt1*), and those involved in the production of reactive nitrogen and oxygen species (e.g., *Nos2*, *Duox1/2*) (Fig 1B). Other up-regulated genes were involved in wound repair (e.g., Amphiregulin, *Areg* [17]) and have been recently implicated in CRC cell proliferation, such as *Steap4* [30] and *Lrg1* [31]. Notably, however, no cell cycle genes were induced by 3 hours, and many metabolic pathways were down-regulated, including the Aryl hydrocarbon receptor (AhR) pathway, recently implicated in maintaining the intestinal stem cell niche [32], and cellular lipid metabolism. We also did not find up-regulation of genes previously implicated in “cancer stemness,” such as *Nanog*, *Sox2*, or *Dot1l* [8]. However, we did detect some overlap with genes that have also been found to be up-regulated by IL-22 in colonic organoids [28]. Notable exceptions were genes involved in retinoic acid metabolism and vasculature development, which were not enriched in small intestine organoids (S1C and S1D Fig). In summary, the main role of IL-22 in the small intestine appears to be protection from infection, through synthesis of antimicrobial proteins, increased mucus production, and oxidative stress.

### Defective IL-22–mediated gene regulation in APC-mutant intestinal epithelial cells

**Fig 2:**
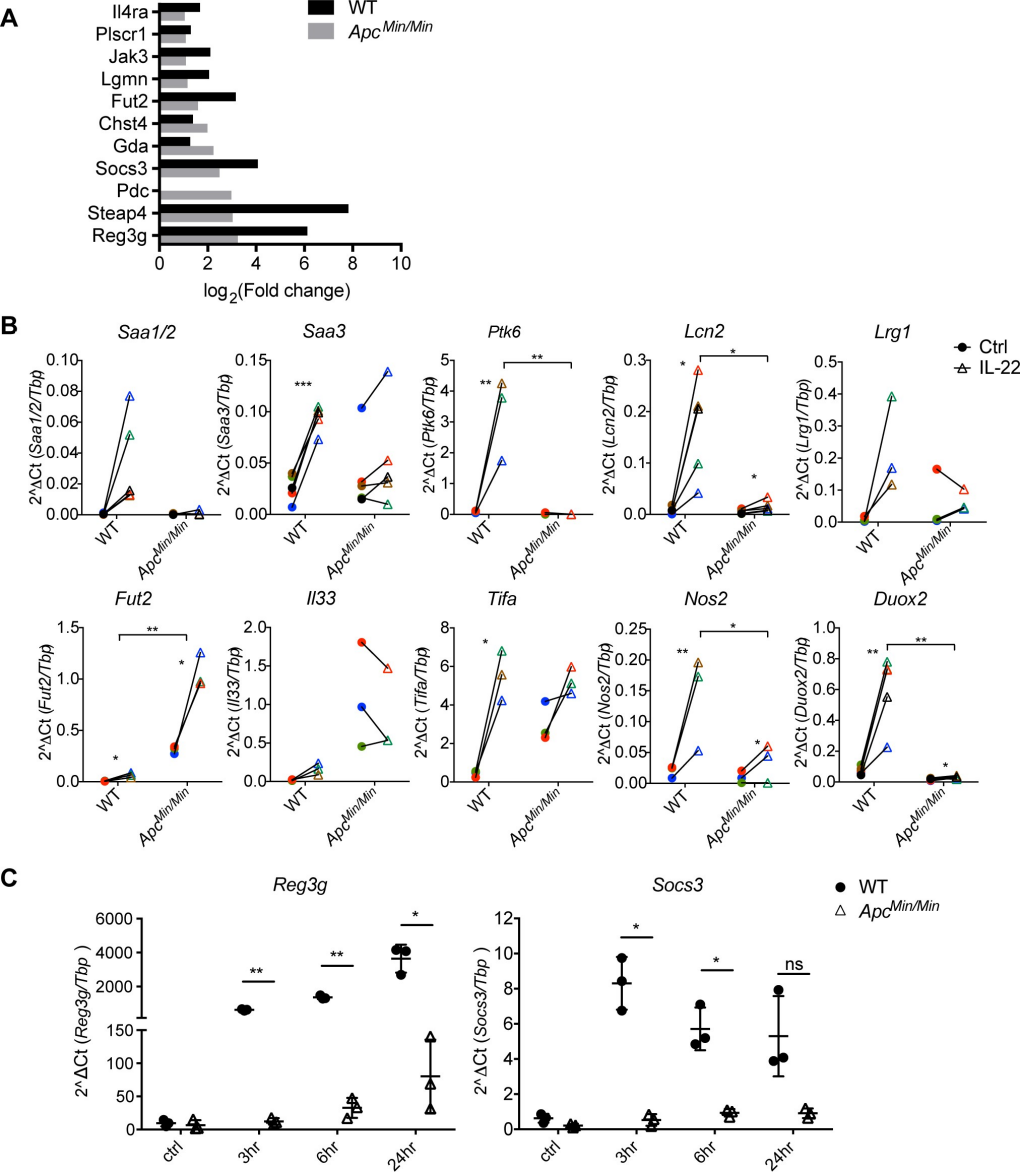
APC-mutant intestinal epithelial cells are defective in IL-22 mediated gene regulation. (A). WT and *Apc*^*Min/Min*^ organoids were treated with IL-22 (2 ng/ml) for 3 hours. RNA was isolated for RNAseq analysis. Graph shows the fold change of genes induced by IL-22 in WT and *Apc*^*Min/Min*^ organoids. The 11 genes shown were the only genes up-regulated by IL-22 in *Apc*^*Min/Min*^ organoids (*p* < 0.1). (B) Expression of candidate up-regulated genes from RNAseq data were verified by RT-qPCR. Data shown are relative levels of mRNA for genes of interest relative to *Tbp* expression. At least 3 independent experiments were performed in each case. *p < 0.05, **p < 0.01, ***p < 0.001 on paired *t* test. (C). WT and *Apc*^*Min/Min*^ organoids were treated with IL-22 (10 ng/ml) for 3, 6, or 24 hours, and RT-qPCR analysis was performed. Data show the relative expression of mRNA for *Reg3g* or *Socs3* compared to *Tbp*. Three independent experiments were performed. *p < 0.05, **p < 0.01, paired *t* test. Numerical values for (A), (B), and (C) are available in S1 Data. APC, adenomatous polyposis coli; IL-22, interleukin-22; *Reg3g*, regenerated islet-derived protein 3 gamma; RNAseq, RNA sequencing; RT-qPCR, quantitative reverse transcription polymerase chain reaction; *Socs3*, Suppressor of cytokine signalling 3; *Tbp*, TATA box binding protein; WT, wild-type.

Because IL-22 has been implicated in promoting stemness and proliferation in CRC cell lines [8,15–17], we asked how transformed small intestinal epithelial cells responded to IL-22. We treated transformed *Apc*^*Min/Min*^ small intestine organoids [26] derived from *Apc*^*Min/+*^ mice with IL-22 and performed RNAseq. In contrast to WT cells, we found only a minute number of genes regulated by IL-22 in APC-mutant intestinal epithelial cell, and these were only detectable when reducing the stringency of the *p*-value threshold to *p* < 0.1 (S1A Fig). Ten of the 11 up-regulated genes were also induced by IL-22 in WT cells, indicating that IL-22 did not activate a different gene transcription programme in transformed cells (Fig 2A). The nonresponsiveness of *Apc*^*Min/Min*^ organoids to IL-22 was confirmed by RT-qPCR analysis (Fig 2B). Note that even at later time points, the gene transcription response to IL-22 was similarly reduced (Fig 2C), confirming that IL-22 did not activate a gene transcription programme in *Apc*^*Min/Min*^ organoids.

### Transformed intestinal epithelial cells respond poorly to IL-22 due to specific down-regulation of the IL-22 receptor

**Fig 3:**
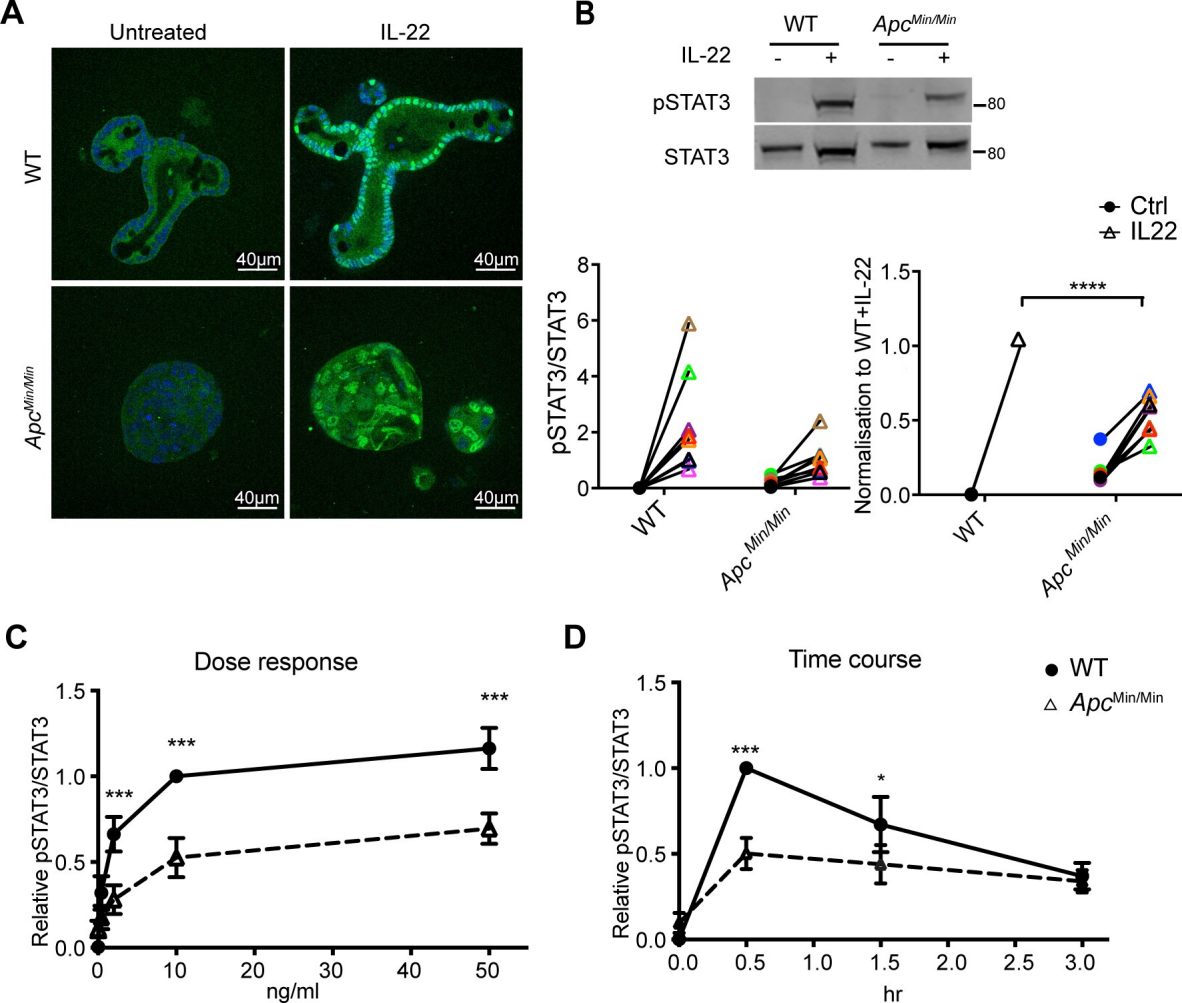
Differences in IL-22–induced STAT3 phosphorylation in WT and *Apc*^*Min/Min*^ organoids. (A). WT and *Apc*^*Min/Min*^ organoids were stimulated with IL-22 (10 ng/ml) for 0.5 hours. Organoids were fixed and immunostained with STAT3 antibodies (green), and nuclei were visualised with Hoechst (blue). (B). Immunoblots and corresponding quantification of pSTAT3 (Tyr705) in WT and *Apc*^*Min/Min*^ organoids with or without IL-22 stimulation. Relative level is expressed as the ratio of pSTAT3 (Tyr705) to total STAT3 in each sample and normalised to the corresponding ratio in WT organoids treated with IL-22 in each experiment. (C) Dose response of pSTAT3 (Tyr705) in organoids treated with 0.4, 2, 10, or 50 ng/ml IL-22 for 0.5 hours. (D). Time course of pSTAT3 (Tyr705) in organoids stimulated with IL-22 (10 ng/ml) for 0.5, 1, or 3 hours. pSTAT3/STAT3 levels in panels C and D were quantified by immunoblotting and normalised to WT + IL-22 at 10 ng/ml IL-22 and at 0.5 hours, respectively. *p < 0.05, **p < 0.01, ***p < 0.001, two-way ANOVA. Numerical values for (B), (C), and (D) can be found in S1 Data. Apc, adenomatous polyposis coli; IL-22, interleukin-22; pSTAT3, phosphorylated STAT3; STAT3, signal transducer and activator of transcription 3; Tyr705, Tyrosine 705; WT, wild-type.

To understand the almost complete lack of response to IL-22 in *Apc*^*Min/Min*^ organoids, we investigated the activation of components of the IL-22 signalling pathway. The most prominent effect of IL-22 in epithelial cells is activation of Janus Kinase 1/Tyrosine kinase 2 (JAK1/Tyk2)-mediated phosphorylation of STAT3 at Tyrosine 705 to regulate gene expression [33]. We first measured STAT3 expression by flow cytometry and confirmed that WT and *Apc*^*Min/Min*^ organoids have similar levels of STAT3 (S2A Fig). We then stimulated organoids with IL-22 and measured STAT3 phosphorylation at the activating residue Tyrosine 705 (pSTAT3) by immunostaining. IL-22 induced robust STAT3 phosphorylation in both WT and *Apc*^*Min/Min*^ organoids (Fig 3A) with corresponding accumulation of pSTAT3 in the nucleus. However, consistently lower levels of pSTAT3 were induced in *Apc*^*Min/Min*^ organoids compared to WT organoids (approximately 50% lower, Fig 3B). Moreover, this reduced response to IL-22 was neither improved after longer times nor was it improved when higher doses of IL-22 were used (Fig 3C and 3D). Phos-tag gels confirmed that a smaller proportion of the total STAT3 was phosphorylated in response to IL-22 in *Apc*^*Min/Min*^ organoids (S2B Fig).

**Fig 4:**
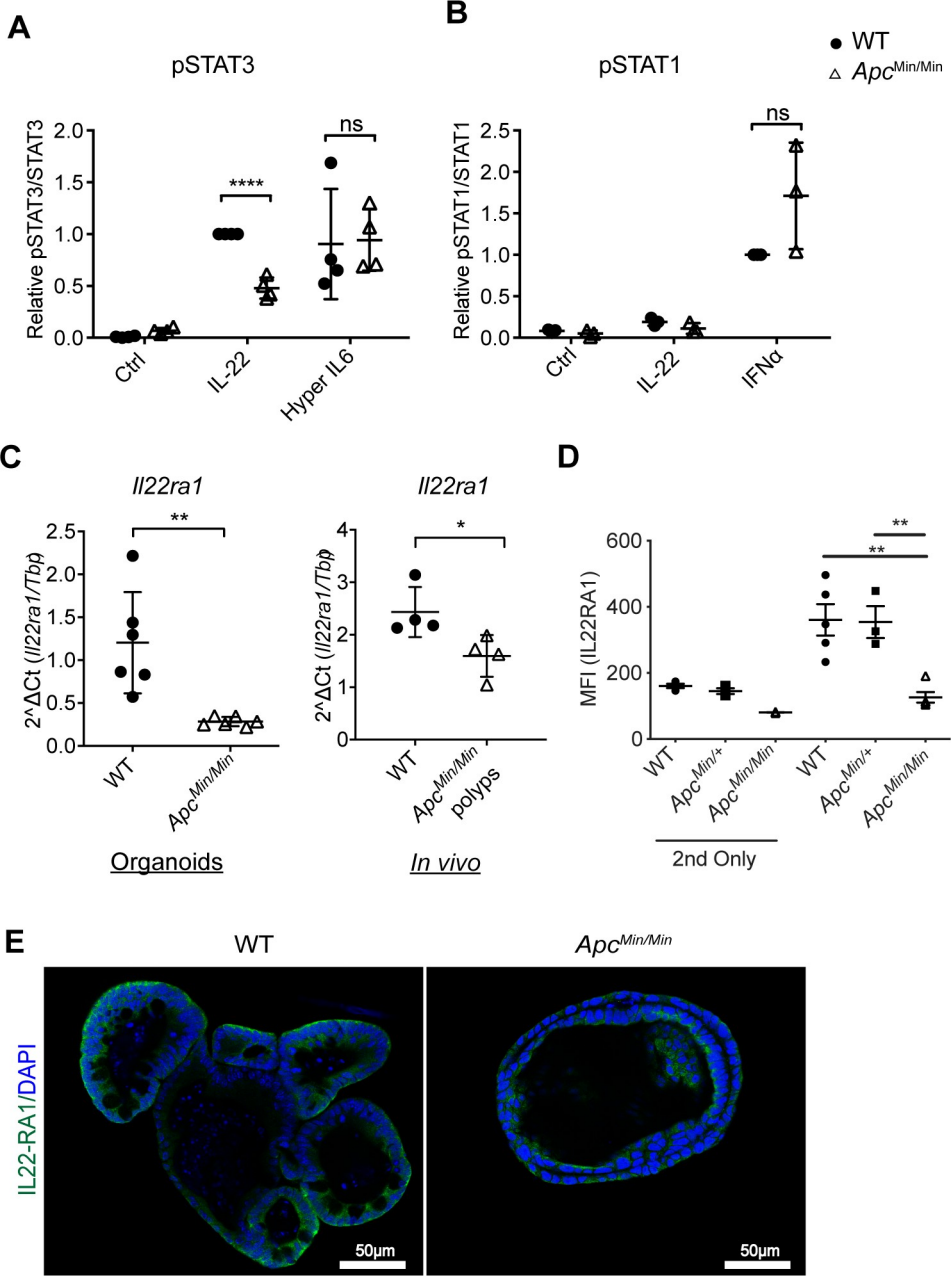
*Apc*^*Min/Min*^ organoids lose expression of the IL-22 receptor. (A) Amount of pSTAT3 (Tyr705)/STAT3 in WT and *Apc*^*Min/Min*^ organoids stimulated with IL-22 (10 ng/ml) or hyper IL-6 (50 μM) for 0.5 hours, normalised to that in WT treated with IL-22 in each experiment. **p < 0.01, paired *t* test. (B) pSTAT1 (Tyr701)/STAT1 in WT and *Apc*^*Min/Min*^ organoids untreated or stimulated with IL-22 or IFNα (1,000 U/ml) for 0.5 hours, normalised to that in WT cells treated with IFNα in each experiment. (C) RT-qPCR analysis of *Il22ra1* expression in WT and *Apc*^*Min/Min*^ organoids, and in tissue biopsies (*in vivo*) from WT intestines or polyps from *Apc*^*Min/+*^ mice. Organoids, *n* = 6, tissue, *n* = 3. *p < 0.05, two-tailed *t* test. (D) WT, *Apc*^*Min/+*^, or *Apc*^*Min/Min*^ organoids were dissociated, and IL22RA1 expression was measured by flow cytometry. MFI of IL22RA1 staining from 3 biological replicates is plotted. “2nd only” reflects samples that were stained only with secondary antibody (i.e., not exposed to primary antibodies). **p < 0.01 on one-way ANOVA. (D) WT and *Apc*^*Min/Min*^ organoids were fixed and immunostained with IL22RA1 antibodies (green), and nuclei were stained with Hoechst (blue). Numerical values for (A), (B), (C), and (D) are available in S1 Data. Apc, adenomatous polyposis coli; IFN, interferon; IL-22, interleukin-22; IL22RA1, interleukin 22 receptor subunit alpha 1; MFI, mean fluorescence intensity; pSTAT3, phosphorylated STAT3; STAT1/3, signal transducer and activator of transcription 1/3; Tyr705, Tyrosine 705; RT-qPCR, quantitative reverse transcription polymerase chain reaction; WT, wild-type.

These data indicated that transformed *Apc*^*Min/Min*^ intestinal epithelial cells are intrinsically defective in activating JAK1/Tyk2-mediated phosphorylation of STAT3 in response to IL-22. To determine whether this effect was specific to IL-22, we tested whether other cytokines also produced reduced activation of STATs in *Apc*^*Min/Min*^ compared to WT organoids. IL-6 increases pSTAT3 in intestinal epithelial cells [34], and interferon (IFN)α activates STAT1 phosphorylation [35]. We treated organoids with the designer cytokine hyper IL-6 (a fusion of the soluble IL-6 receptor [sIL-6R] with IL-6), which bypasses the requirement for the IL-6 receptor α chain to directly activate the gp130 receptor [36], or with IFNα. We found that hyper IL-6 induced pSTAT3 (Fig 4A) and IFNα induced pSTAT1 (Fig 4B) similarly in WT and *Apc*^*Min/Min*^ organoids (S2D Fig). Therefore, the reduced levels of pSTAT3 in *Apc*^*Min/Min*^ organoids are specific to IL-22 responses.

These results suggest that the reduction in pSTAT3 levels in *Apc*^*Min/Min*^ cells in response to IL-22 was caused by differences upstream of STAT activation, at the level of the cytokine receptor. Indeed, our RNAseq data revealed that mRNA for both IL-22 receptor subunits—*Il22ra1* and *Il10rb*—and the signalling components *Jak1* and *Tyk2* were lower in *Apc*^*Min/Min*^ than in WT organoids (S3A and S3B Fig). Further analysis by RT-qPCR confirmed that *Apc*^*Min/Min*^ intestinal epithelial cells have significantly lower levels of *Il22ra1* mRNA both in organoids and in polyps in vivo (Fig 4C). We also detected reduced cell surface and total IL-22 receptor expression on *Apc*^*Min/Min*^, but not on heterozygous *Apc^Min/+^*, intestinal epithelial cells (Fig 4D and 4E). This could explain the lower induction of pSTAT3 specifically in response to IL-22 in APC-mutant cells, because gp130 expression (*il6st*) was not down-regulated (S3A Fig).

### Elevated levels of HDACs in APC-mutant intestinal epithelial cells inhibit STAT3 target gene transcription

**Fig 5:**
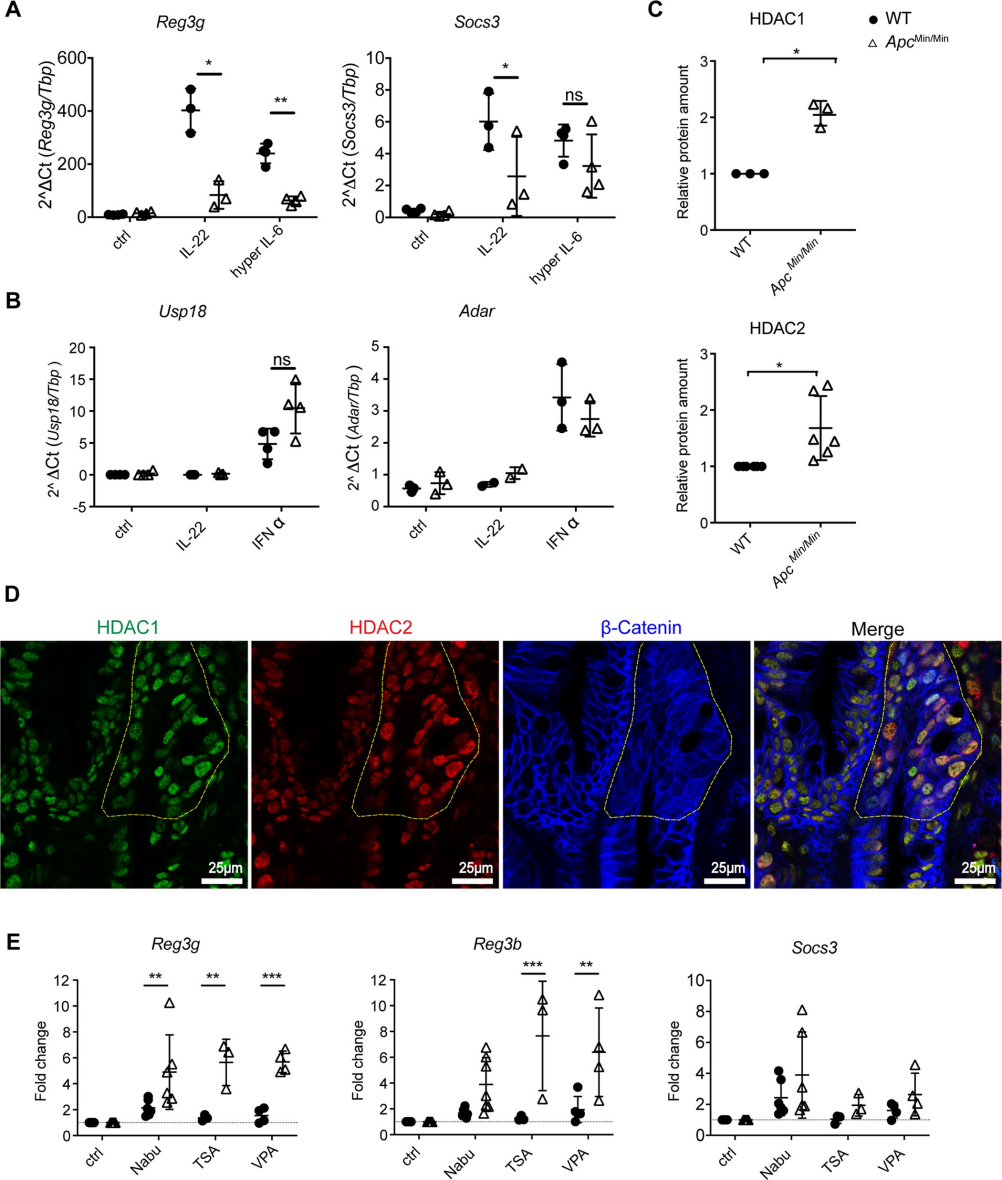
HDAC inhibition partially rescues STAT3-mediated gene transcription in *Apc*^*Min/Min*^ organoids. (A) RT-qPCR analysis of WT and *Apc*^*Min/Min*^ organoids treated with IL-22 (10 ng/ml) or hyper IL-6 (50 μM) for 3 hours. Data show mRNA expression of STAT3 target genes, *Reg3g* or *Socs3*, relative to *Tbp*. (B) RT-qPCR analysis for WT and *Apc*^*Min/Min*^ organoids treated with IL-22 (10 ng/ml) or IFNα (1,000 U/ml) for 3 hours. Data show the expression of STAT1 target genes, *Adar* and *Usp18* relative to *Tbp*. At least 3 independent experiments were performed. *p < 0.05, **p < 0.01, paired *t* test. (C) Western blot analysis of proteins in WT and *Apc*^*Min/Min*^ organoids. Expression of HDAC1 and HDAC2 was normalized to loading control, STAT3, in each sample and expressed relative to that in WT organoids. (D) *Apc*^*Min/+*^ mouse small intestine was fixed and stained with antibodies against HDAC1, HDAC2, and β-catenin. Nuclei were visualised with DAPI. *Apc*^*Min/Min*^ small intestinal polyps (marked by yellow dotted line) were defined by diffuse and increased β-catenin staining. (E) WT and *Apc*^*Min/Min*^ organoids were treated with NaBu (10 mM), TSA (50 nM), or VPA (1 mM) for 16 hours and then stimulated with IL-22 (10 ng/ml) for 3 hours. RT-qPCR was performed. Data show the expression of *Reg3g*, *Reg3b*, and *Socs3* relative to the IL-22–stimulated control in each genotype. **p < 0.01, ***p < 0.005, Two-way ANOVA, with Sidak’s multiple comparison. At least 4 independent experiments were performed. Numerical values for (A), (B), (C), and (E) are available in S1 Data. *Adar*, Adenosine deaminase RNA specific; Apc, adenomatous polyposis coli; HDAC, histone deacetylase; IFN, interferon; IL-22, interleukin-22; NaBu, sodium butyrate; ns, not significant; RT-qPCR, quantitative reverse transcription polymerase chain reaction; *Reg3g*, regenerated islet-derived protein 3 gamma; *Reg3b*, regenerated islet-derived protein 3 beta; *Socs3*, Suppressor of cytokine signalling 3; STAT3, signal transducer and activator of transcription 3; TSA, Trichostatin A; *Usp18*, ubiquitin specific peptidase 18; VPA, valproic acid; WT, wild-type.

Hyper IL-6 induced pSTAT3 to similar levels in WT and *Apc*^*Min/Min*^ intestinal epithelial cells. Nonetheless, induction of the STAT3 target gene *Reg3g* was still lower in *Apc*^*Min/Min*^ cells in response to IL-6 compared to WT, although *Socs3* was equivalently induced (Fig 5A). On the other hand, IFNα-STAT1 target genes were equivalently induced in WT and *Apc*^*Min/Min*^ cells (Fig 5B). This confirmed that transcriptional activation for some STAT3 target genes was compromised in *Apc*^*Min/Min*^ intestinal epithelial cells.

Transcription by STAT3 is regulated by a number of factors. In addition to phosphorylation at Tyrosine 705, IL-22 increases the phosphorylation of STAT3 at Serine 727, a site required for maximal STAT3 transcriptional activation [37]. In contrast to pSTAT3 (Tyrosine 705) induction, which was impaired in *Apc*^*Min/Min*^ cells, we did not observe a difference in the level of IL-22–induced pSTAT3 (Serine 727) in WT and *Apc*^*Min/Min*^ organoids (S2C Fig). Once STAT3 is activated, it recruits coactivators such as Nuclear receptor coactivator 1 (NCOA1) or CREB binding protein (CBP)/p300 to the promoter of a target gene. In addition, its activity can also be negatively regulated by type I HDACs, protein inhibitor of activated STAT3 (PIAS3), and suppressor of cytokine signaling 3 (SOCS3) [38–42]. Our RNAseq data revealed lower gene expression of the STAT3 coactivator *Ncoa1* in APC-mutant cells and higher expression of *Hdac1/2* (S3B Fig), but not of any of the other coregulators. We also detected higher protein expression of HDAC1 and HDAC2 in *Apc*^*Min/Min*^ organoids (Fig 5C). Moreover, higher HDAC1 and HDAC2 expression was observed in *Apc*^*Min/Min*^ small intestinal polyps compared with *Apc*^*Min/+*^ cells in vivo (Fig 5D). To further determine whether elevated HDACs contributed to lower expression of STAT3 target genes in APC mutant cells, we measured whether inhibition of HDACs by NaBu, TSA, and VPA could enhance STAT3 transcriptional activity. RT-qPCR results showed that 3 diverse HDAC inhibitors had a stronger effect on *Apc*^*Min/Min*^ than WT organoids in restoring gene expression in response to IL-22 (Fig 5E). Regardless, HDAC inhibition still did not restore IL-22–mediated gene expression in *Apc*^*Min/Min*^ organoids to the level in WT organoids (S3D Fig). This is consistent with our finding that *Apc*^*Min/Min*^ cells express less IL-22R and that IL-22 treatment cannot activate pSTAT3 to the same level as in WT organoids, due to many defects in the IL-22 signalling pathway (S3 Fig). It is also noteworthy that some genes, including *Socs3* (Fig 5E), were minimally affected by HDAC inhibition, suggesting that these genes were not regulated by HDACs. Thus, the defective response of transformed intestinal epithelial cells to IL-22 is potentially a combination of reduced expression of the IL-22 receptor and high levels of HDACs expressed in *Apc*^*Min/Min*^ cells.

### Adenomas in vivo are also defective in responding to IL-22

**Fig 6:**
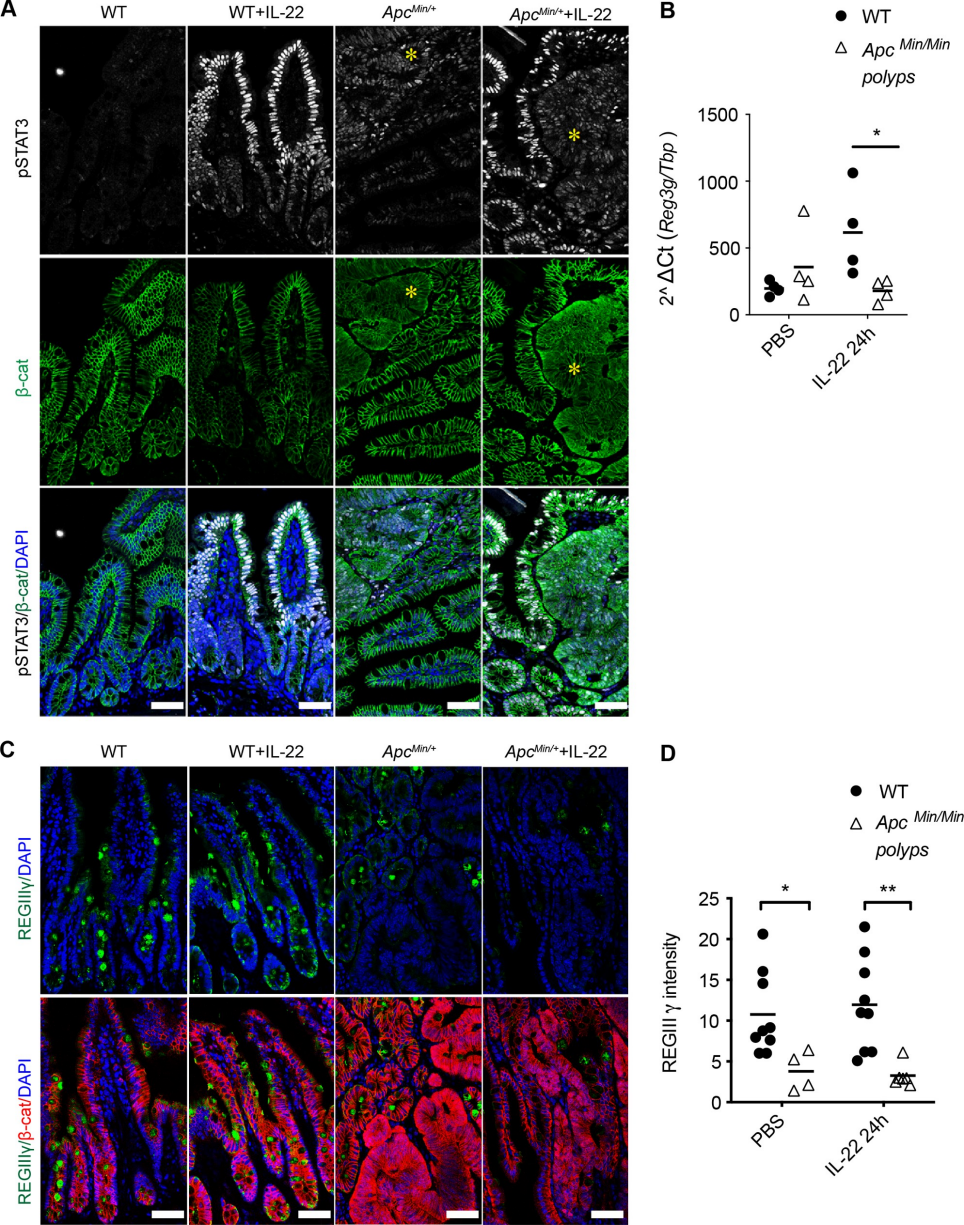
Reduced IL-22 responses in cells in *Apc*^*Min/Min*^ small intestinal polyps in vivo. Mice aged between 88 and 95 days were injected i.p. with 1 μg IL-22 or PBS. (A) Small intestine was harvested after 1 hour and tissue stained with antibodies against pSTAT3 (Tyr705) and β-catenin. Nuclei were stained with DAPI. Asterisk indicate polyps. (B) Small intestine was harvested 24 hours after IL-22 injection. RNA was isolated, and RT-qPCR was performed. Data show the level of *Reg3g* mRNA relative to *Tbp*. *P < 0.05, two-way ANOVA. (C) Small intestine was harvested 24 hours after IL-22 injection, and IHC was performed using antibodies against RegIIIγ and β-catenin. Nuclei were stained with DAPI. Three independent experiments were performed. Number of mice used in total: WT = 3; *Apc*^*Min/+*^ = 6. Scale bar = 50 μm. (D) Mean fluorescence intensity for RegIIIγ in the epithelial area analysed using Fuji software. Each dot represents the mean fluorescence intensity in 1 confocal image. *p < 0.05, **p < 0.01, *t* test. Numerical values for (B) and (D) are available in S1 Data. β-cat, β-catenin; Apc, adenomatous polyposis coli; DAPI, 4',6-diamidine-2'-phenylindole dihydrochloride; IHC, immunohistochemistry; IL-22, interleukin-22; i.p., intraperitoneal; pSTAT3, phosphorylated signal transducer and activator of transcription 3; *Reg3g* or RegIIIγ, Regenerated islet-derived protein 3 gamma; RT-qPCR, quantitative reverse transcription polymerase chain reaction; Tyr705, Tyrosine 705; WT, wild type.

To test whether the differential response to IL-22 also existed in vivo, we injected WT or *Apc*^*Min/+*^ mice with IL-22 and measured STAT3 phosphorylation. In WT intestinal epithelia, pSTAT3 expression was not observed in PBS-injected control. Upon IL-22 treatment, nuclear pSTAT3 was strongly induced in WT intestinal tissue. In *Apc*^*Min/+*^ mice, IL-22 also induced pSTAT3 nuclear accumulation in untransformed cells. In polyps, which are composed of homozygous *Apc*^*Min/Min*^ cells, a weak signal for nuclear pSTAT3 was already detectable prior to IL-22 treatment. However, there was no significant increase of pSTAT3 in polyps after injecting *Apc*^*Min/+*^ mice with IL-22 (Fig 6A). Note that *Apc*^*Min/Min*^ tissue/polyps are identifiable by diffuse and increased β-catenin staining, confirming that the cells they contain had undergone LOH. In contrast, in *Apc*^*Min/+*^ heterozygous tissue, β-catenin localized to the lateral membranes, and only these cells exhibited strong nuclear pSTAT3 after IL-22 injection. These data confirm that epithelial cells heterozygous for APC mutation respond to IL-22 similarly to WT cells, whereas homozygous mutant *Apc*^*Min/Min*^ cells do not respond to IL-22 in vivo.

We next asked whether the lack of pSTAT3 induction resulted in lower STAT3 target gene expression in *Apc*^*Min/Min*^ polyps. *Reg3g* was one of the most highly induced antimicrobial genes in response to IL-22 in organoids. Consistent with our in vitro data, *Reg3g* mRNA was induced in response to IL-22 in WT, but not in *Apc*^*Min/Min*^ tissue (Fig 6B). We also observed significantly lower expression of RegIIIγ peptides in polyps compared to WT or *Apc*^*Min/+*^ epithelia with or without IL-22 injection (Fig 6C and 6D). Thus, the loss of IL-22 responsiveness in homozygous *Apc*^*Min/Min*^ cells was also recapitulated in vivo.

### IL-22 induces oxidative stress and promotes early intestinal tumorigenesis

**Fig 7:**
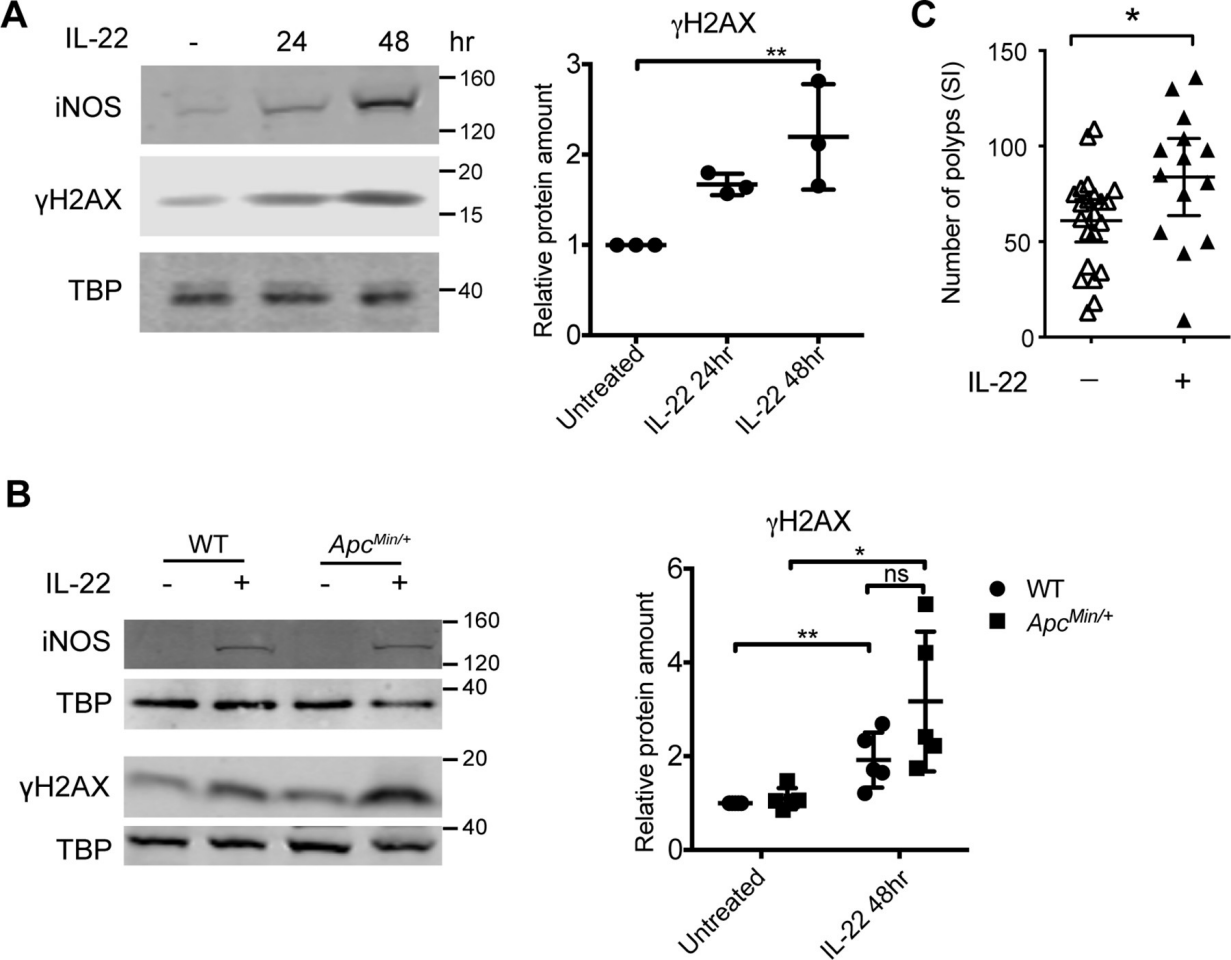
IL-22 increases iNOS and DNA damage in *Apc*^*Min/+*^ organoids. (A) Western blot of iNOS or γH2AX in WT organoids treated with IL-22 (10 ng/ml) for 24 or 48 hours. (B) Western blotting for iNOS or γH2AX in WT or *Apc*^*Min/+*^ organoids treated with IL-22 (10 ng/ml) for 48 hours. TBP was used as loading control. The ratio of γH2AX to TBP in each sample was normalised to untreated control in each experiment. At least 3 independent experiments were performed. *p < 0.05, paired *t* test. (C) *Apc*^*Min/+*^ mice aged 27–30 days were injected twice a week for 4 weeks with 1 μg IL-22 or PBS or were left untreated. Untreated and PBS-injected mice were pooled together for the “control” cohort (there was no difference in the tumour number in these two cohorts). *p < 0.05, unpaired *t* test. Numerical values for (A) and (B) are available in S1 Data. γH2AX, Phospho-Histone H2A.X (Serine 139); Apc, adenomatous polyposis coli; IL-22, interleukin-22; iNOS, inducible nitric oxide synthase; SI, small intestine; TBP, TATA box binding protein; WT, wild-type.

Since transformed *Apc*^*Min/Min*^ tissue responded poorly to IL-22, we reasoned that IL-22 must contribute to intestinal tumorigenesis by acting on *Apc*^*Min/+*^ cells, before LOH drives loss of IL-22 responses. Many inflammatory cytokines induce production of reactive oxygen and nitrogen species (RONS) as part of the defence response. Importantly, these free radicals can also cause DNA damage. We hypothesized that IL-22–induced oxidative stress could promote tumorigenesis by driving DNA damage. Our RNAseq data indicated that IL-22 up-regulated the iNOS *Nos2* and Dual oxidases (*Duox1/2*) (Figs 1C and 2C), as well as several genes involved in DNA damage (e.g., *Parp9*, *Gadd45g*). We confirmed that IL-22 increased *Nos2* and *Duox2* mRNA in WT cells (S4A Fig). Moreover, IL-22 induced iNOS protein in both WT and heterozygous *Apc*^*Min/+*^ cells (Fig 7A and 7B). The DNA damage marker γH2AX was also increased by IL-22 in both WT and *Apc*^*Min/+*^ cells (Fig 7A and 7B and S4B Fig). This suggested that IL-22–induced DNA damage could contribute to LOH and initiate APC-mutation–dependent intestinal transformation.

To test this idea, we injected young (4- to 5-week-old) *Apc*^*Min/+*^ mice with low levels (1 μg) of IL-22 twice a week for 4 weeks, to mimic underlying inflammation. Indeed, after 5 weeks, we found more polyps in the small intestine of *Apc*^*Min/+*^ mice that had been injected with IL-22 than in animals that had not been injected with IL-22 (Fig 7C). These observations support the idea that IL-22 could drive tumorigenesis by promoting LOH in *Apc*^*Min/+*^ cells in mice.

## Discussion

The cytokine IL-22 has emerged as an important player in intestinal tumorigenesis. IL-22 can increase cell proliferation [15] and enhance cancer stemness by increasing expression of core stem cell genes [8], but it can also control elimination of damaged intestinal stem cells, preventing tumorigenesis [18]. We specifically investigated the mechanism by which IL-22 contributes to small intestinal tumorigenesis in the *Apc*^*Min/+*^ mouse model for cancer. Unexpectedly, we found that transformed *Apc*^*Min/Min*^ cells in both organoids and in animals responded poorly to IL-22. We further showed that this was likely due to low IL-22 receptor expression and reduced STAT3 transcriptional activity in *Apc*^*Min/Min*^ cells, although we cannot eliminate additional causes. Our data suggest that rather than contributing to intestinal tumorigenesis by augmenting the proliferation of transformed cells, IL-22 acts at an early stage to drive genetic instability in *Apc*^*Min/+*^ cells, thus increasing the occurrence of transforming LOH events.

A significant prediction of our work is that the absence of IL-22 responses in *Apc*^*Min/Min*^ cells may lead to increased intestinal inflammation due to reduced immune defence of the intestinal barrier. Previous reports and our own transcriptome analysis show that the main function of IL-22 is to up-regulate defence and immune responses [14,28,43], through up-regulation of mucus production, mucus glycosylation, and antimicrobial peptide production. IL-22–producing ILCs can prevent bacterial translocation across the intestinal barrier. Correspondingly, lack of IL-22, or IL-22 receptor, contributes to a heightened inflammatory milieu in the intestinal epithelium, as well as increased sensitivity to bacterial infection due to increased systemic dissemination of bacteria [28,44,45]. In this context, previous studies showed compromised intestinal barrier integrity and increased inflammation in *Apc*^*Min/+*^ and *Apc*^*Floxed/wt*^;*Cdx2-Cre* mice [46,47]. Consistent with the lack of IL-22 responses in *Apc*^*Min/Min*^ cells, we found that the antimicrobial peptide RegIIIγ was nearly undetectable in polyps in *Apc*^*Min/+*^ mice. Lack of RegIIIγ can result in increased bacterial translocation across the intestinal epithelial barrier, and the decreased mucosal protection in *Reg3g*^*−/−*^ mice correlates with increased inflammation [44,45]. Thus, the lack of RegIIIγ expression in polyps, even in the presence of IL-22, may cause impaired barrier functions and increased inflammation in *Apc*^*Min/Min*^ polyps, which may contribute to tumour formation.

In the absence of IL-22 responses in transformed *Apc*^*Min/Min*^ cells, one likely explanation for the tumour-promoting effect of IL-22 in *Apc*^*Min/+*^ mice is provided by our discovery that IL-22 induces expression of iNOS and Duox1/2. This is predicted to result in increased RONS and DNA damage [1,13]. Consistent with the idea that RONS could accelerate polyp formation, inhibition of nitric oxide production reduced tumour loads in *Apc*^*Min/+*^ mice [48]. In addition, studies in a colitis-associated carcinogenesis mouse model using *H*. *hepaticus* infection showed that IL-22 could induce DNA damage via a nitric oxide–dependent mechanism to promote dysplasia [49]. Infection of 4-week-old *Apc*^*Min/+*^ mice with *Citrobacter rodentium*, a colonic attaching and effacing pathogen that induces a potent IL-22 response, increased colonic tumour burden [50]. We observed increased DNA damage in response to IL-22 as evidenced by γH2AX induction. We also observed increased small intestinal tumour burden upon multiple IL-22 injections into young preneoplastic *Apc*^*Min/+*^ mice. Together, these data support the idea that IL-22 has mutagenic effects on nontransformed cells, mediated by RONS. This, in turn, could explain the reduction in tumour numbers in *Il22*^−/−^ *Apc*^*Min/+*^ compared to *Il22*^*+/+*^ *Apc*^*Min/+*^ mice.

Here, we focus on addressing why *Il22*^−/−^*Apc*^*Min/+*^ mice develop fewer small intestinal polyps than *Il22*^*+/+*^*Apc*^*Min/+*^mice [16]. However, in studies of colonic carcinogenesis [16,18], *Il22*^−/−^ mice develop more tumours under inflammation-inducing or mutagenic conditions. This suggests that the effects of IL-22 are different in the small intestine and colon. Indeed, our comparison of IL-22–induced genes in the small intestine with colonic gene expression data support this conclusion. Interestingly, we find that IL-22 induces γH2AX expression in small intestinal epithelial cells, and it was recently shown that induction of γH2AX in colonic crypts after irradiation damage was also strictly dependent on IL-22 expression [18]. Moreover, IL-22 increases proliferation in colonic and small intestinal stem cells [15], and this increased proliferation may also increase the frequency of LOH events in *Apc*^*Min/+*^mice. Together, these studies imply that IL-22 may have opposing functions in intestinal tumorigenesis, dependent on the underlying cause of carcinogenesis.

It is intriguing that APC-mutant cells selectively down-regulate IL-22–dependent pSTAT3 signalling, whereas IL-6–induced pSTAT3 is unaffected. One explanation for the lower IL-22R expression could be reduced stability of the protein. The IL-22R is phosphorylated by glycogen synthase kinase 3 (GSK3), which prevents its proteasomal degradation [51]. In the presence of mutant APC, GSK3 activity is reduced [52], which may partially explain the low surface expression of the IL-22 receptor in *Apc*^*Min/Min*^ cells. However, it should be noted that our RNAseq data indicated differential expression of nearly 5,000 genes in *Apc*^*Min/Min*^ compared to WT organoids, suggesting that there are multiple possible reasons for the loss of IL-22 responsiveness in *Apc*^*Min/Min*^ cells. For example, we also found decreased expression of key IL-22 signal transducing elements—*Il10rb*, *Jak1*, and *Tyk2—*in *Apc*^*Min/Min*^ cells (S4 Fig). On the other hand, we also clearly show increased HDAC1/2 expression in *Apc*^*Min/Min*^ cells, and inhibition of HDACs in *Apc*^*Min/Min*^ organoids had a strong effect on IL-22 target gene expression. Therefore, multiple pathways contribute to the loss of IL-22 responses in *Apc*^*Min/Min*^ cells.

Mutations in APC are found in up to 80% of nonhereditary CRCs [20]. However, we are not suggesting that all transformed epithelial cells cannot respond to IL-22. When analysing a panel of human cell lines, we found that a subset of them did not respond to IL-22, and some of these had reduced expression of the IL-22 receptor [3]. On the other hand, high levels of IL-22 have been found in many CRC samples and are associated with CRC development [4,5,17]. Whether the presence of IL-22 is important at an early stage for the development of tumours, or for maintaining tumour progression, is not yet clearly established. Regardless, our data advocate exercising caution when considering IL-22 as a therapeutic target for treatment of established CRC and suggest that patients need to be stratified based on expression of the IL-22 receptor, STAT3 repressors, and possibly other mutations acquired by cancerous cells.

## Supporting information

S1 Fig Transcriptome comparison of IL-22 responses in WT small intestinal and colonic organoids. (A) Representative image of WT organoids on day 3 of culture. (B) Table indicating the number of genes significantly regulated by IL-22 in WT or *Apc*^*Min/Min*^ organoids and the thresholds used to filter the data. (C) Heatmap clustering of differentially expressed genes in WT organoids treated with or without IL-22 for 3 hours in 3 biological replicates. Heatmap: red = higher expression, blue = lower expression, relative to the row mean expression of the gene across all samples. (D) Out of the top 50 IL-22–up-regulated genes in colonic organoids [28] only 29 had a fold change > 2 in IL-22–treated small intestinal organoids. (E) The top 20 biological processes (filtered, GO_BP_FAT) regulated by IL-22 in colonic and small intestinal organoids. Numerical values for (E) are available in S1 Data. FC, fold change.
(TIF)

S2 Fig IL-22 induces phosphorylation of STAT3 (at Tyrosine 705 and Serine 727). (A) Flow cytometric analysis of STAT3 expression in WT and *Apc*^*Min/Min*^ organoids. (B) Phos-tag gels were used to separate phosphorylated and nonphosphorylated STAT3. Immunoblot for STAT3 shows nonphosphorylated (lower band) and phosphorylated (upper band) STAT3 protein. The same membrane was incubated with anti-pSTAT3 (Tyrosine 705) to confirm the identity of the upper band as pSTAT3. Plot shows the percentage of total STAT3 that is phosphorylated. (C) Western blot analysis shows pSTAT3 (Serine 727) levels in WT and *Apc*^*Min/Min*^ organoids with or without IL-22 stimulation (10 ng/ml) for 0.5 hours. Data show the ratio of pSTAT3 (Serine 727) to total STAT3 in each sample normalised to that in WT organoids treated with IL-22 in each experiment. (D) Representative western blot of pSTAT3 (Tyrosine 705), STAT3, pSTAT1 (Tyrosine 701), or STAT1 in WT and *Apc*^*Min/Min*^ organoids treated with IL-22 (10 ng/ml), hy-IL6 (50 μM), or IFNα (1,000 U/ml) for 0.5 hours. Numerical values for (B) and (C) are available in S1 Data. hy-IL6, hyper IL-6
(TIF)

S3 Fig *Apc*^*Min/Min*^ organoids express lower mRNA levels of IL-22 signalling pathway genes. RNAseq data for mRNA levels of (A) *Il22ra1*, *Il10rb*, and *Il6st*; (B) *Jak1*, *Tyk2*, and *Stat3*; and (C) *Hdac1*, *Hdac2*, and *Ncoa1* in WT and *Apc*^*Min/Min*^ organoids. **P < 0.01, ***P < 0.001, and ****P < 0.0001, by two-tailed *t* test. (D) WT and *Apc*^*Min/Min*^ organoids were pretreated with HDAC inhibitors NaBu, TSA, and VPA for 16 hours before stimulation with IL-22 (10 ng/ml) for 3 hours. All 3 inhibitors partially rescued expression of *Reg3g* and *Reg3b* in *Apc*^*Min/Min*^ organoids, although the expression was not restored to WT levels. Data from 4–7 independent biological replicates are shown. Numerical values for (A), (B), (C), and (D) are available in S1 Data. RPKM, reads per kilobase per million mapped reads
(TIF)

S4 Fig IL-22 increases expression of Nos2, Duox2, and DNA damage in WT organoids. (A) RT-qPCR analysis of WT organoids treated with IL-22 (10 ng/ml) for 3, 24, or 48 hours. Data show the mRNA expression of *Reg3g*, *Nos2*, *Duox2*, *Atm*, *Atr*, and *Lgr5*, relative to *Tbp*. At least 3 independent experiments were performed. *P < 0.05 **P < 0.01 and ***P < 0.001 by one-way ANOVA, using Geisser-Greenhouse correction. (B) WT organoids were treated with IL-22 (10 ng/ml) for 48 hours. Organoids were fixed and stained with γH2AX antibodies (green). Nuclei were stained with DAPI (blue). Numerical values for (A) are available in S1 Data.
(TIF)

S1 Table Sequences of primers used for RT-qPCR. (DOCX)

S2 Table Annotated RNAseq data comparing WT organoids treated with IL-22 versus untreated. (XLSX)

S3 Table Annotated RNAseq data comparing *Apc*^*Min/Min*^ organoids treated with IL-22 versus untreated. (XLSX)

S4 Table Annotated RNAseq data comparing *Apc*^*Min/Min*^ organoids versus WT organoids. (XLSX)

S5 Table Annotated RNAseq data comparing *Apc*^*Min/Min*^ organoids treated with IL-22 versus WT organoids treated with IL-22. (XLSX)

S1 Data Data underlying Figs 1B, 2A, 2B, 2C, 3B, 3C, 3D, 4A, 4B, 4C, 4D, 5A, 5B, 5C, 5E, 6B, 6D, 7A, 7B, 7C, S1E, S2B, S2C, S3A, S3B, S3C, S3D and S4A. (XLSX)

S1 Raw Images Raw images of western blotting data included in Figs 3B, 7A and 7B, S2B, S2C and S2D. (PDF)

## Abbreviations

γH2AX: Phospho-Histone H2A.X
ADF: Advanced DMEM/F12
AhR: Aryl hydrocarbon receptor
Apc: Adenomatous polyposis coli gene
CBP: CREB binding protein
CRC: colorectal cancer
DAPI: 4',6-diamidine-2'-phenylindole dihydrochloride
Duox: dual oxidase
FAP: familial adenomatous polyposis
GSK3: Glycogen synthase kinase 3
HDAC: histone deacetylase
IFNα: interferon α
IHC: immunohistochemistry
IL-10R2: interleukin 10 receptor 2
IL-22: interleukin-22
IL-22RA1: interleukin 22 receptor subunit alpha 1
ILC: innate lymphoid cells
iNOS: inducible nitric oxide synthase
JAK1: Janus Kinase 1
LOH: loss of heterozygosity
Min: multiple intestinal neoplasia
NaBu: sodium butyrate
Ncoa1: Nuclear receptor coactivator 1
PIAS3: protein inhibitor of activated STAT3
pSTAT3: phosphorylated STAT3
RegIIIγ or Reg3g: Regenerated islet-derived protein 3 gamma
RNAseq: RNA sequencing
RONS: reactive oxygen species and nitrogen species
RPKM: Reads Per Kilobase of exon per Million reads mapped
RT-qPCR: quantitative reverse transcription polymerase chain reaction
sIL-6R: soluble IL-6 receptor
SOCS3: Suppressor of cytokine signalling 3
STAT3: signal transducer and activator of transcription 3
Tbp: TATA box binding protein
TSA: Trichostatin A
Tyk: Tyrosine kinase 2
VPA: valproic acid
WT: wild-type
