# Supplementary figures and images for: Loss of adenomatous polyposis coli function renders intestinal epithelial cells resistant to the cytokine IL-22
Source: PLoS Biol. 2019 Nov 26;17(11):e3000540. doi: 10.1371/journal.pbio.3000540 (PMC6903767; doi:10.1371/journal.pbio.3000540)

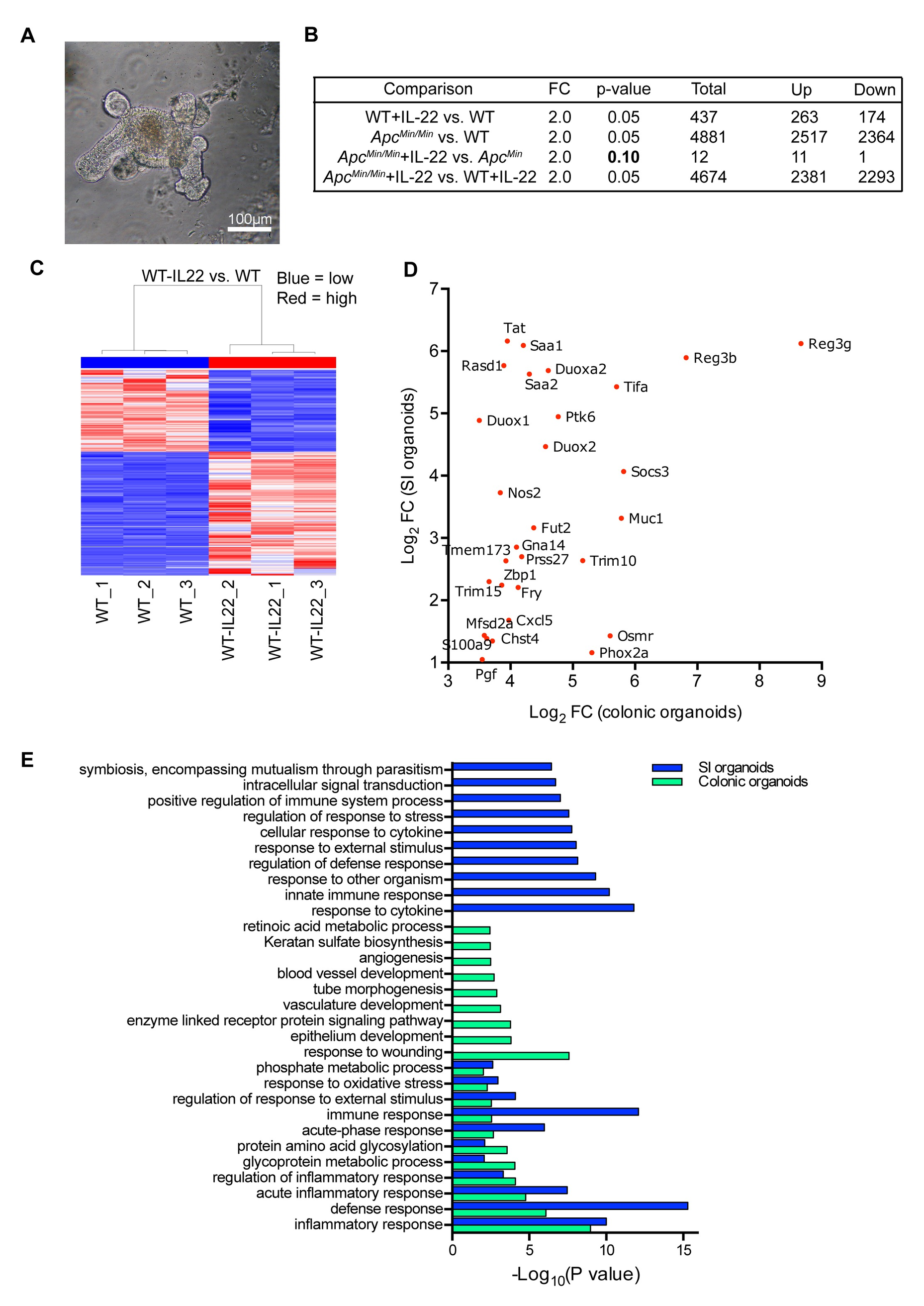

Supplement: S1 Fig — (A) Representative image of WT organoids on day 3 of culture. (B) Table indicating the number of genes significantly regulated by IL-22 in WT or ApcMin/Min organoids and the thresholds used to filter the data. (C) Heatmap clustering of differentially expressed genes in WT organoids treated with or without IL-22 for 3 hours in 3 biological replicates. Heatmap: red = higher expression, blue = lower expression, relative to the row mean expression of the gene across all samples. (D) Out of the top 50 IL-22–up-regulated genes in colonic organoids [28] only 29 had a fold change > 2 in IL-22–treated small intestinal organoids. (E) The top 20 biological processes (filtered, GO_BP_FAT) regulated by IL-22 in colonic and small intestinal organoids. Numerical values for (E) are available in S1 Data. FC, fold change. (TIF) [file pbio.3000540.s001.tif]

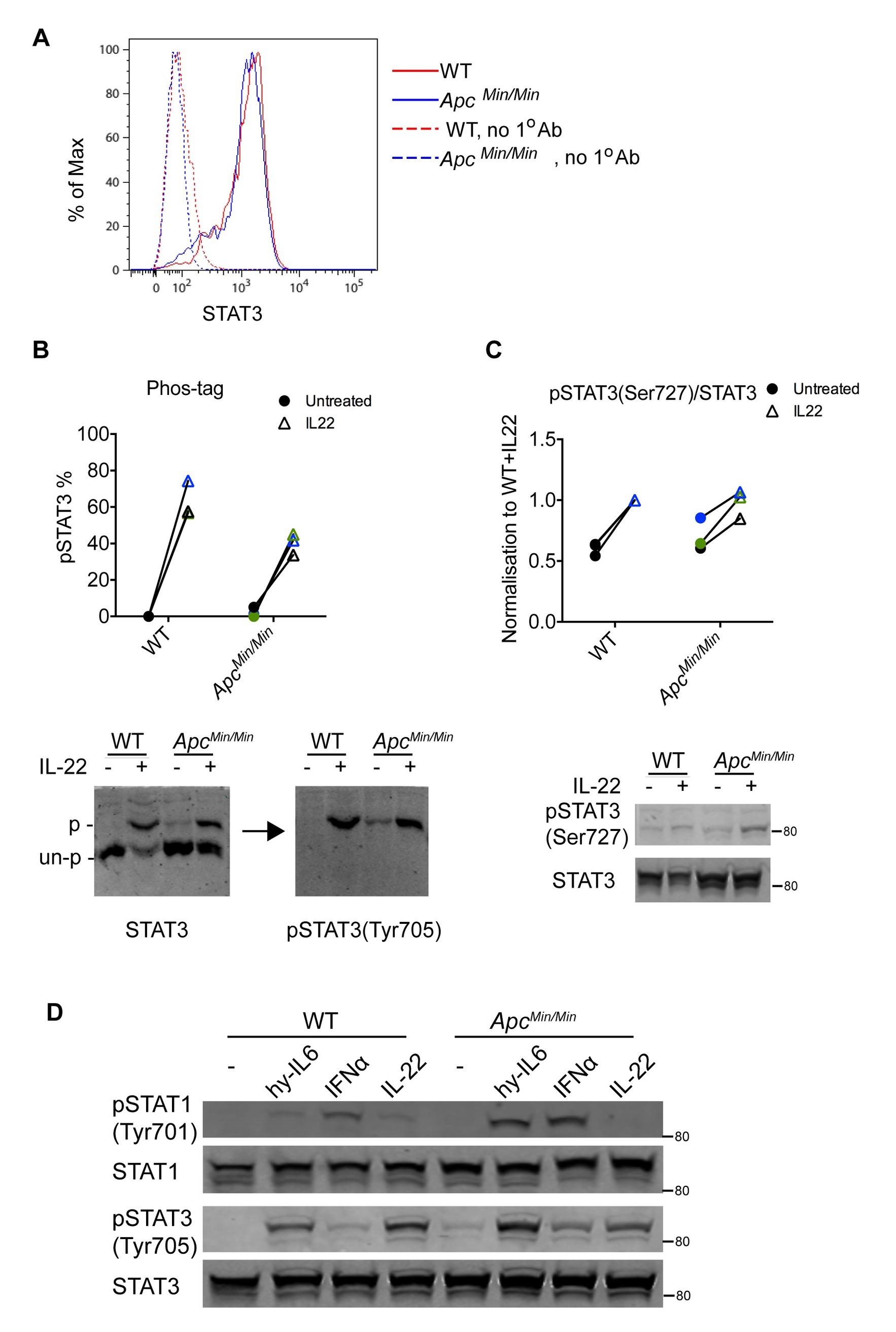

Supplement: S2 Fig — (A) Flow cytometric analysis of STAT3 expression in WT and ApcMin/Min organoids. (B) Phos-tag gels were used to separate phosphorylated and nonphosphorylated STAT3. Immunoblot for STAT3 shows nonphosphorylated (lower band) and phosphorylated (upper band) STAT3 protein. The same membrane was incubated with anti-pSTAT3 (Tyrosine 705) to confirm the identity of the upper band as pSTAT3. Plot shows the percentage of total STAT3 that is phosphorylated. (C) Western blot analysis shows pSTAT3 (Serine 727) levels in WT and ApcMin/Min organoids with or without IL-22 stimulation (10 ng/ml) for 0.5 hours. Data show the ratio of pSTAT3 (Serine 727) to total STAT3 in each sample normalised to that in WT organoids treated with IL-22 in each experiment. (D) Representative western blot of pSTAT3 (Tyrosine 705), STAT3, pSTAT1 (Tyrosine 701), or STAT1 in WT and ApcMin/Min organoids treated with IL-22 (10 ng/ml), hy-IL6 (50 μM), or IFNα (1,000 U/ml) for 0.5 hours. Numerical values for (B) and (C) are available in S1 Data. hy-IL6, hyper IL-6 (TIF) [file pbio.3000540.s002.tif]

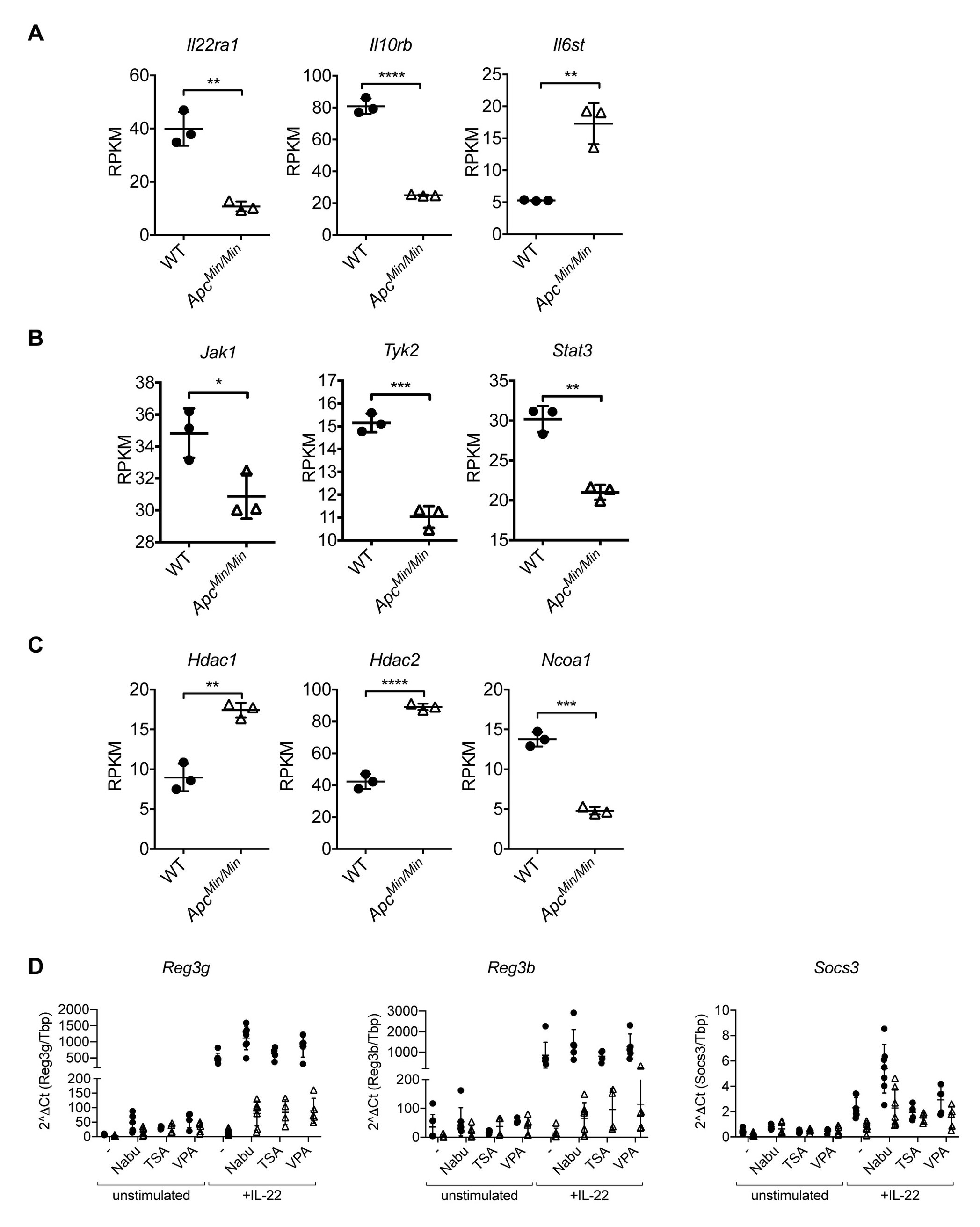

Supplement: S3 Fig — RNAseq data for mRNA levels of (A) Il22ra1, Il10rb, and Il6st; (B) Jak1, Tyk2, and Stat3; and (C) Hdac1, Hdac2, and Ncoa1 in WT and ApcMin/Min organoids. **P < 0.01, ***P < 0.001, and ****P < 0.0001, by two-tailed t test. (D) WT and ApcMin/Min organoids were pretreated with HDAC inhibitors NaBu, TSA, and VPA for 16 hours before stimulation with IL-22 (10 ng/ml) for 3 hours. All 3 inhibitors partially rescued expression of Reg3g and Reg3b in ApcMin/Min organoids, although the expression was not restored to WT levels. Data from 4–7 independent biological replicates are shown. Numerical values for (A), (B), (C), and (D) are available in S1 Data. RPKM, reads per kilobase per million mapped reads (TIF) [file pbio.3000540.s003.tif]

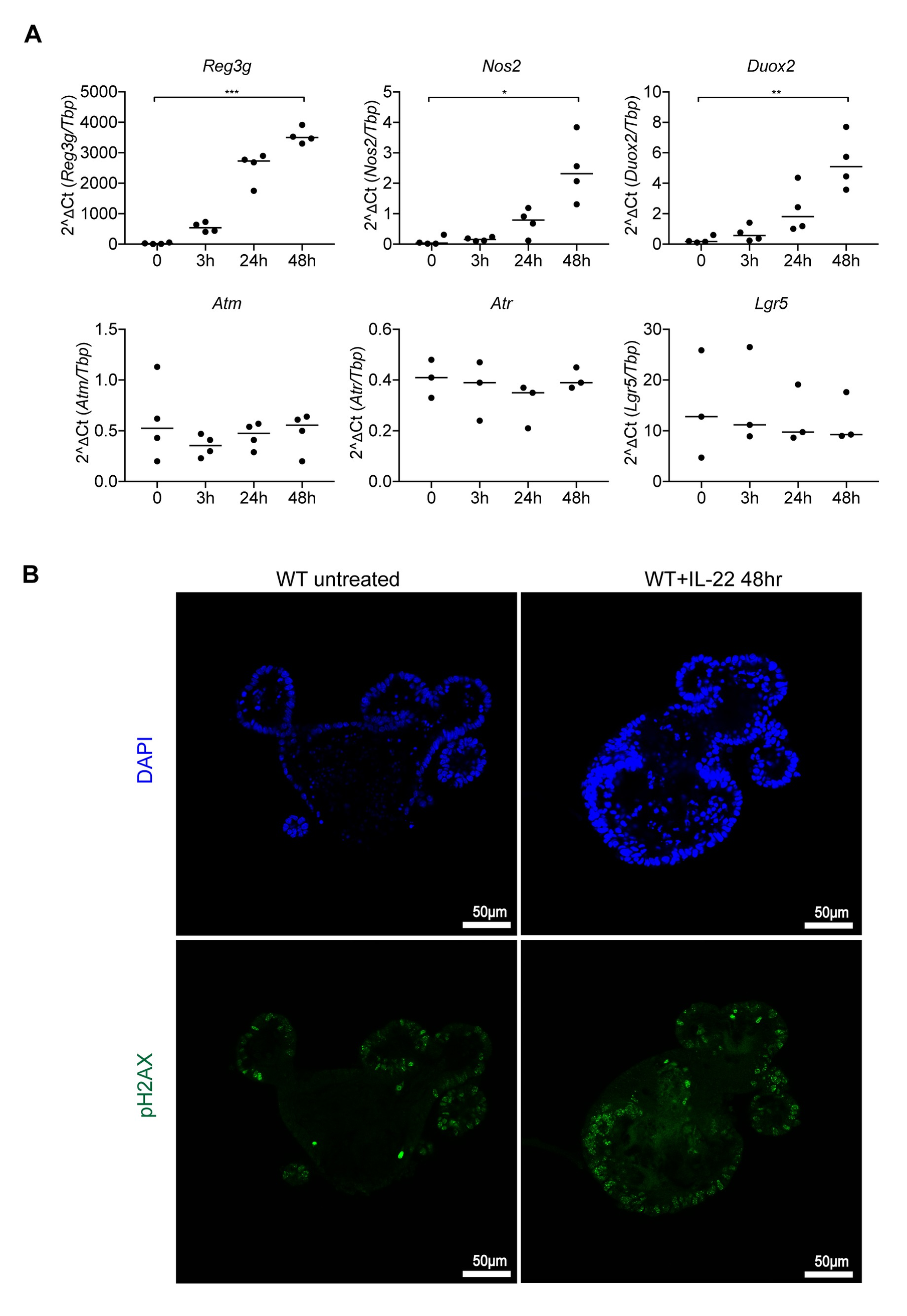

Supplement: S4 Fig — (A) RT-qPCR analysis of WT organoids treated with IL-22 (10 ng/ml) for 3, 24, or 48 hours. Data show the mRNA expression of Reg3g, Nos2, Duox2, Atm, Atr, and Lgr5, relative to Tbp. At least 3 independent experiments were performed. *P < 0.05 **P < 0.01 and ***P < 0.001 by one-way ANOVA, using Geisser-Greenhouse correction. (B) WT organoids were treated with IL-22 (10 ng/ml) for 48 hours. Organoids were fixed and stained with γH2AX antibodies (green). Nuclei were stained with DAPI (blue). Numerical values for (A) are available in S1 Data. (TIF) [file pbio.3000540.s004.tif]

Fig 3B

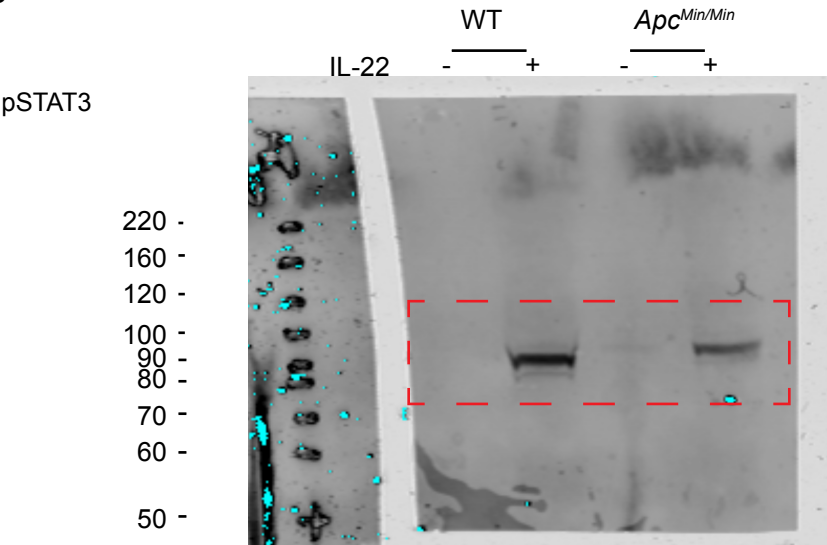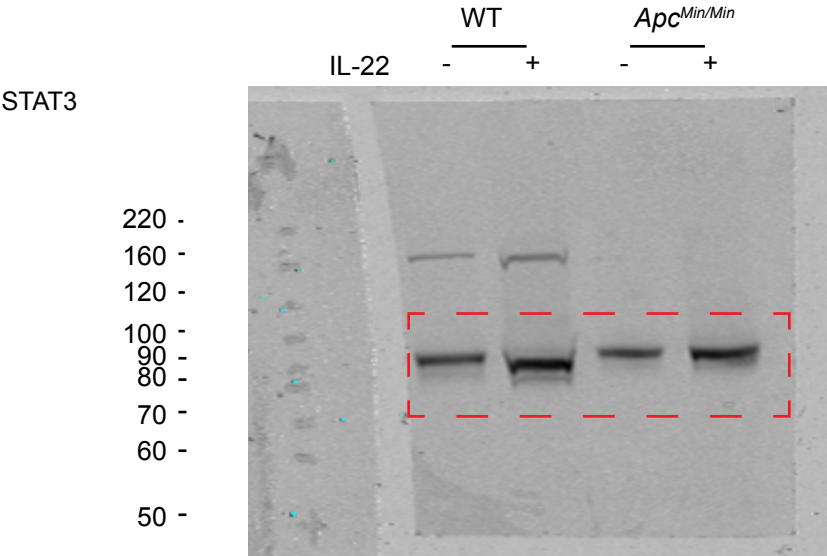

Fig 7A

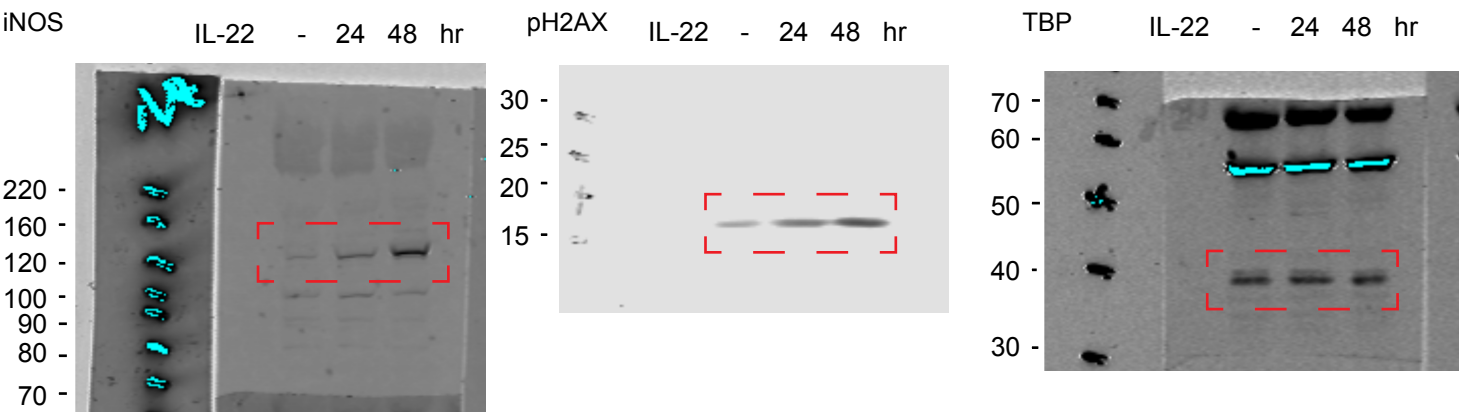

Fig 7B

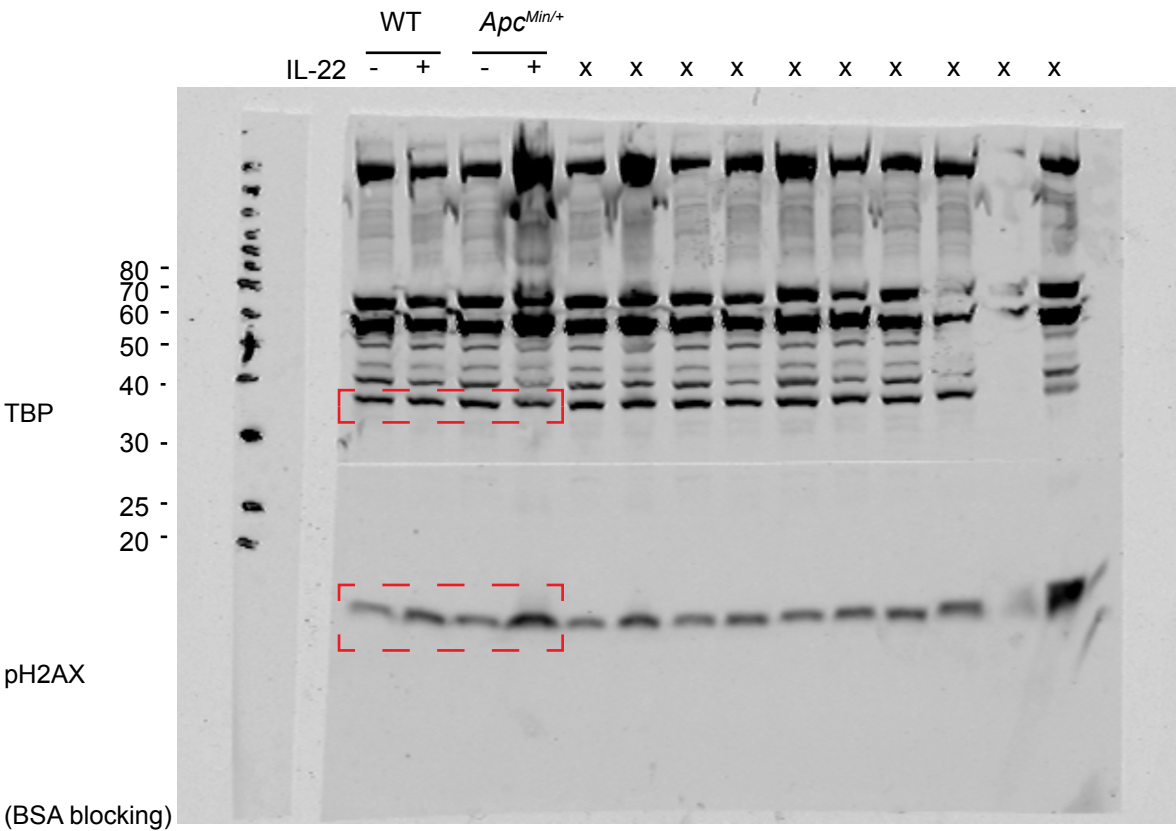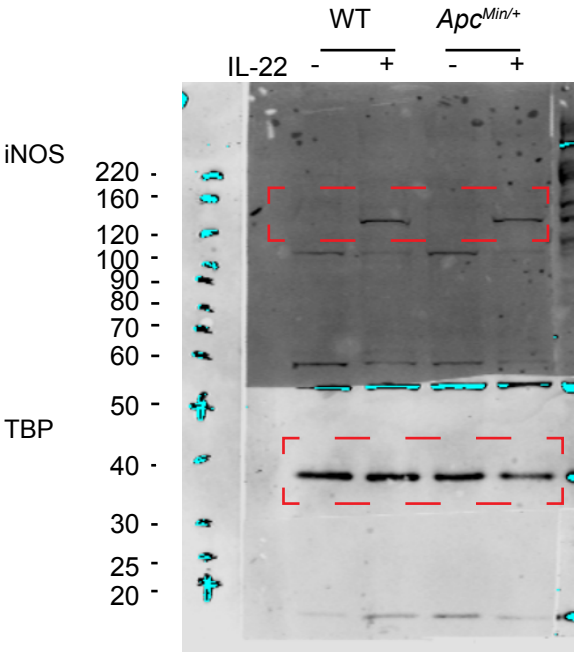

S2B Fig

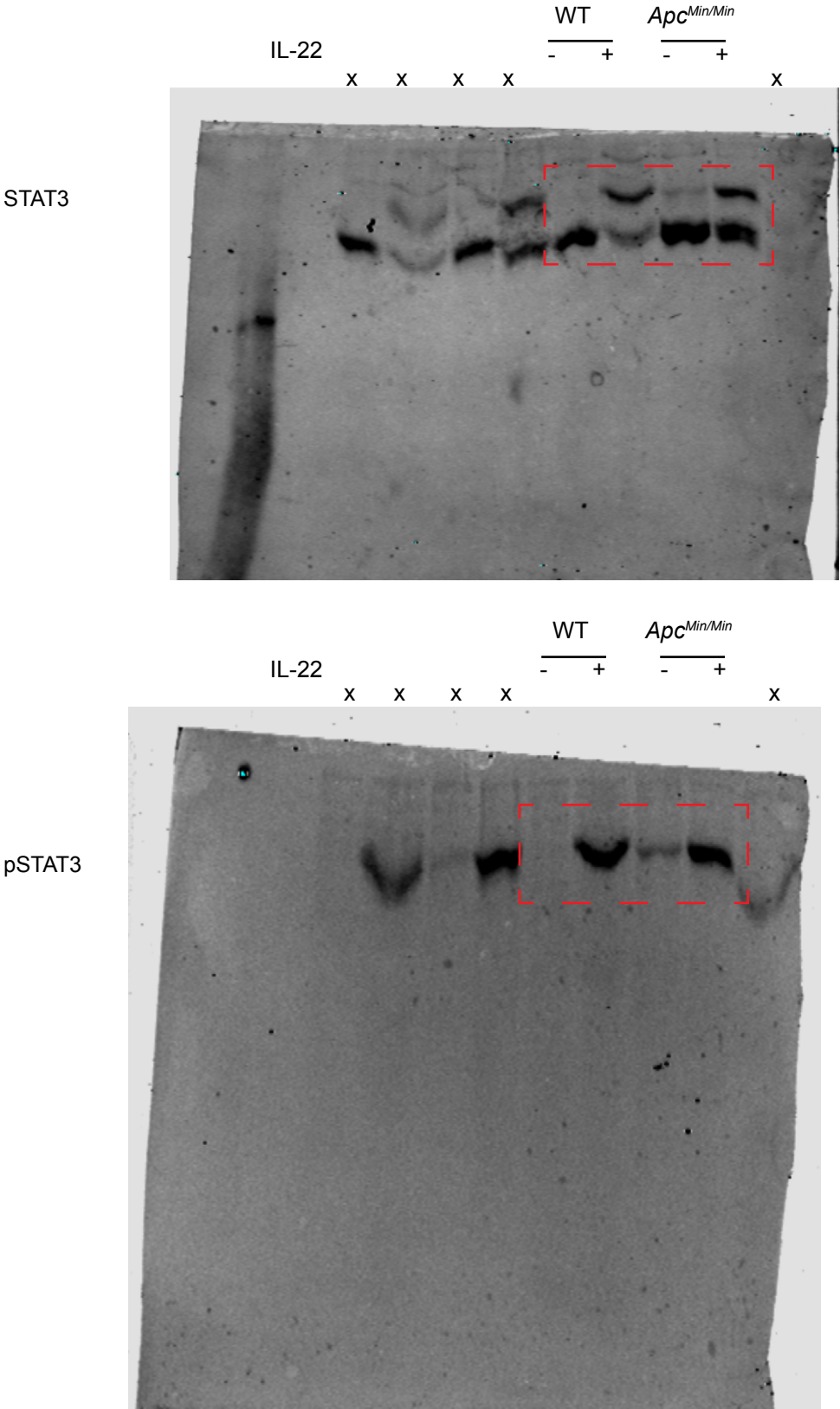

phos-tag gel: molecular weight markers not applicable

S2C Fig

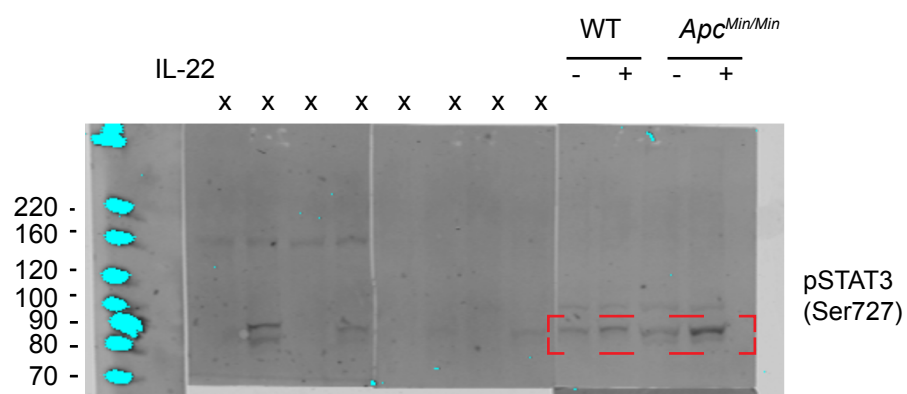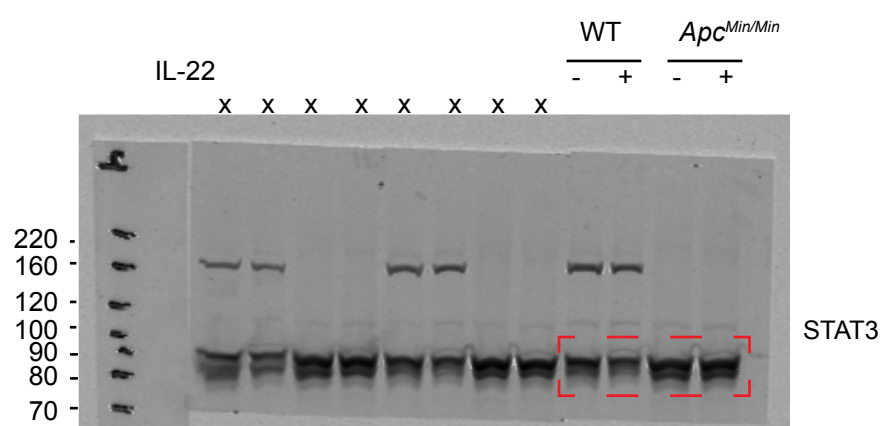

S2D Fig

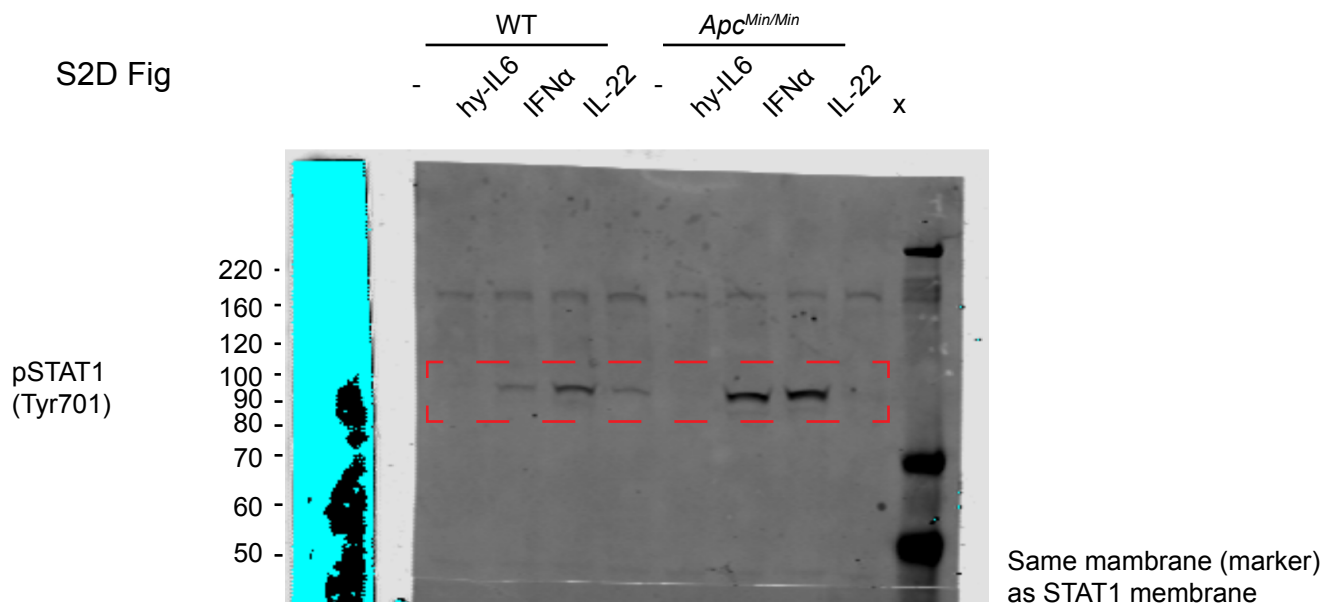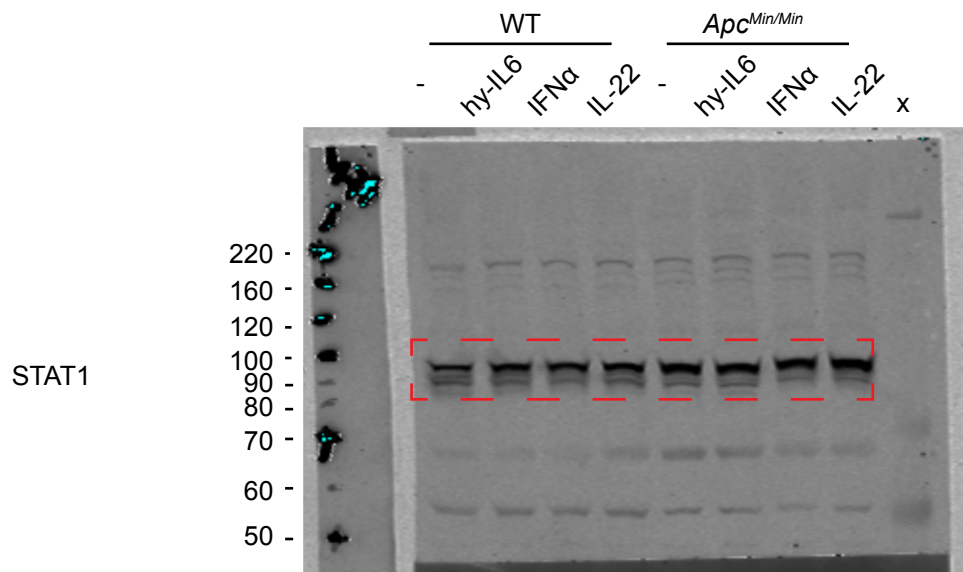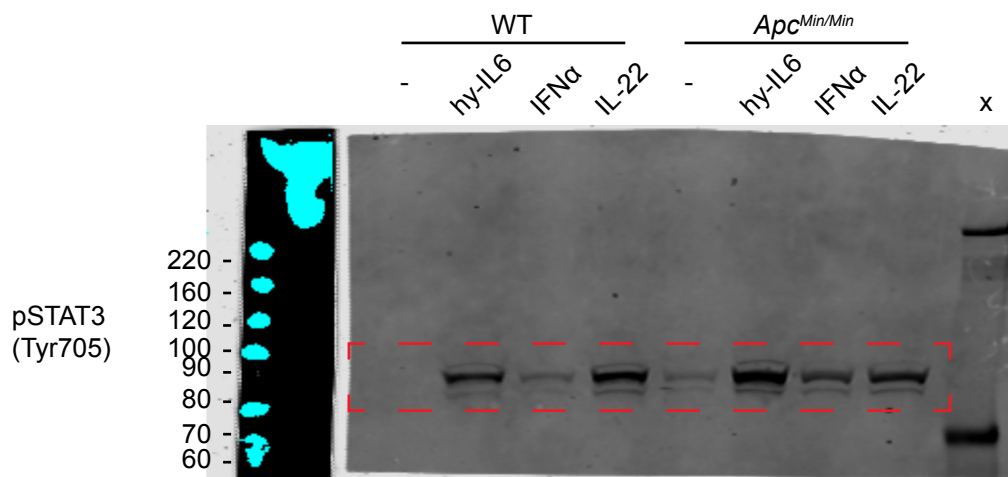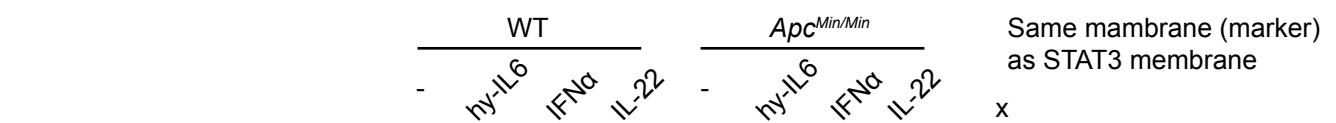

Supplement: S1 Raw Images — (PDF) [file pbio.3000540.s011.pdf]
