# Supplementary material for: Loss of adenomatous polyposis coli function renders intestinal epithelial cells resistant to the cytokine IL-22
Source: PLoS Biol. 2019 Nov 26;17(11):e3000540. doi: 10.1371/journal.pbio.3000540 (PMC6903767; doi:10.1371/journal.pbio.3000540)
Supplement: S1 Table — (DOCX) [file pbio.3000540.s005.docx]

| **Gene** | **Forward primer** | **Reverse primer** |
| --- | --- | --- |
| *Adar* | ggaagaagactcggagaaacc | tcccagagaacaaggatgttg |
| *Atm* | agcttgtgaagggccgtgatga | aacaccgcttcgctgagaaagg |
| *Atr* | agttggccagtgctactccaga | ggtcggctgagcgtcagttttctt |
| *Duox2* | gcactgtgcagaacagctaggacaac | acctcatcaccttcttgcgggag |
| *Fut2* | agagccaagctggaagatacac | atcaaggtggcgtctctctg |
| *Lcn2* | ccatctatgagctacaagagaacaat | tctgatccagtagcgacagc |
| *Lrg1* | ccatgtcagtgtgcagattc | aagagtgagaggtggaagag |
| *Nos2* | ttcagcacatctgcagacac | agcctgaagtcatgtttgcc |
| *Ptk6* | gccgtgcgacattacaggat | taggccatgagacaggctct |
| *Reg3g* | accatcaccatcatgtcctg | ggcatctttcttggcaactt |
| *Saa1/2* | ctgcctgccaaatactgagagtc | ccacttccaagttcctgtttattac |
| *Saa3* | gctggcctgcctaaaagatactg | gcatttcacaagtatttattcagc |
| *Socs3* | gcgggcacctttcttatcc | tccccgactgggtcttgac |
| *Tbp* | ggg­gagctgtgatgtgaagt | ccaggaaataattctggctcat |
| *Tifa* | acgcaattccaacatgtgcc | ccgagctcctggttgtctac |
| *Usp18* | ttgggctcctgaggaaacc | cgatgttgtgtaaaccaaccaga |

**S1 Table**. Sequences of primers used for RT-qPCR
